# Pro-Inflammatory Signaling Upregulates a Neurotoxic Conotoxin-Like Protein Encrypted Within Human Endogenous Retrovirus-K

**DOI:** 10.3390/cells9071584

**Published:** 2020-06-30

**Authors:** Domenico Di Curzio, Mamneet Gurm, Matthew Turnbull, Marie-Josée Nadeau, Breanna Meek, Julia D. Rempel, Samuel Fineblit, Michael Jonasson, Sherry Hebert, Jennifer Ferguson-Parry, Renée N. Douville

**Affiliations:** 1 Department of Biology, University of Winnipeg, 599 Portage Avenue, Winnipeg, MB R3B 2G3, Canada; d.dicurzio@uwinnipeg.ca (D.D.C.); sheena_m17@hotmail.com (M.G.); ave.jor@gmail.com (M.T.); mare.nadeau@gmail.com (M.-J.N.); meek-b@webmail.uwinnipeg.ca (B.M.); julia@sagewaterhealth.com (J.D.R.); sam_fineblit@hotmail.com (S.F.); michael.jonasson@alumni.ubc.ca (M.J.); s.hebert@uwinnipeg.ca (S.H.); jennferguson@gmail.com (J.F.-P.); 2 Department of Immunology, University of Manitoba, 750 McDermot Avenue, Winnipeg, MB R3E 0T5, Canada

**Keywords:** endogenous retrovirus, amyotrophic lateral sclerosis, conotoxin, HIV Tat, NF-κB, necroptosis, myelination

## Abstract

Motor neuron degeneration and spinal cord demyelination are hallmark pathological events in Amyotrophic Lateral Sclerosis (ALS). Endogenous retrovirus-K (ERVK) expression has an established association with ALS neuropathology, with murine modeling pointing to a role for the ERVK envelope (*env*) gene in disease processes. Here, we describe a novel viral protein cryptically encoded within the ERVK *env* transcript, which resembles two distinct cysteine-rich neurotoxic proteins: conotoxin proteins found in marine snails and the Human Immunodeficiency Virus (HIV) Tat protein. Consistent with Nuclear factor-kappa B (NF-κB)-induced retrotransposon expression, the ERVK conotoxin-like protein (CTXLP) is induced by inflammatory signaling. CTXLP is found in the nucleus, impacting innate immune gene expression and NF-κB p65 activity. Using human autopsy specimens from patients with ALS, we further showcase CTXLP expression in degenerating motor cortex and spinal cord tissues, concomitant with inflammation linked pathways, including enhancement of necroptosis marker mixed lineage kinase domain-like (MLKL) protein and oligodendrocyte maturation/myelination inhibitor Nogo-A. These findings identify CTXLP as a novel ERVK protein product, which may act as an effector in ALS neuropathology.

## 1. Introduction

Viruses and toxins are considered potential environmental factors implicated in the motor neuron disease Amyotrophic Lateral Sclerosis (ALS) [1,2,3]. With most ALS cases considered sporadic in nature, the search for gene-environment interactions in ALS has remained disappointingly elusive [4,5]. Endogenous retrovirus-K (ERVK) expression has been repeatedly associated with ALS neuropathology [6,7,8,9], with murine modeling pointing to a role for the ERVK envelope (*env*) gene as a cause of motor neuron loss [10]. However, recent studies conflict with previous findings by suggesting there is no significant difference in ERVK *env* transcripts and protein expression between ALS and control tissues [11,12]. Conversely, ERVK *env* has been shown to be neuroprotective in Human Immunodeficiency Virus (HIV) infection [13]. To date, the role of ERVK *env* in disease remains highly debated, and it has been proposed that proteins other than canonical full-length Env should be considered when examining the role of ERVK in ALS disease [11]. Here, we describe a novel ERVK protein product termed conotoxin-like protein (CTXLP), which is cryptically encoded within ERVK *env* and may help elucidate the neuropathological nature of ERVK reactivation.

ALS pathology involves the degeneration of the upper (brain) and lower (spinal cord) motor neurons, leading to muscle weakness and paralysis (reviewed in [14,15,16]). The majority of ALS cases are considered sporadic, and the cause of this disease remains unknown. Brain and spinal cord inflammation are a hallmark of ALS neuropathology (reviewed in [17,18]). Whether specific viruses have the capacity to cause ALS pathology, or if their role is predominantly as an inflammatory trigger is controversial [2]. Nonetheless, viruses have been repeatedly postulated to play a role in ALS due to the overlapping neuropathology of this disease with several infection models. For instance, renewed interest in the link between enteroviruses and ALS is based on similar cellular and molecular pathology in murine and cell line-based enterovirus infection [1]. As another example, the *env* gene of the murine leukemia virus is a crucial driver of motor neuron disease in mice, albeit with a pathological divergence from what is seen in ALS [19]. In humans, exogenous retroviruses HIV and Human T-cell Lymphotropic Virus (HTLV) are occasionally associated with the development of ALS-like syndrome [20,21,22]. Lastly, endogenous retroviruses, specifically ERVK proviruses, are also suspected of promoting ALS-associated neuropathology [23].

Endogenous retroviruses (ERVs) are viral symbionts that populate the human genome, representing approximately 8% of human genomic DNA [24]. ERVs are found to be highly polymorphic between individuals and different ethnic groups [25]. They can benefit their host [26], or in other contexts, are thought to participate in pathogenesis and disease development [23]. ERVK is the most recently endogenized retrovirus and most biologically active in humans, with approximately 1000 genomic ERVK insertions identified [27]. However, few loci retain the coding capacity for the production of intact viral proteins [28]. ERVK expression has been detected in several tissues throughout the body at varying levels amongst individuals and in a variety of disease states [29]. The role of ERVK in ALS disease pathogenesis remains contentious, intensifying the search for cellular pathways impacted by the ERVK envelope protein.

Unlike their exogenous counterparts, ERVs are often referred to as simple retroviruses. This is because viruses like ERVK encode few regulatory accessory proteins in complement with their core genes [30]. Accessory proteins often modulate cellular pathways; for example, HIV Tat is known to provoke inflammatory responses and neurotoxicity via multiple cellular pathways [31,32]. To search for unaccounted viral and pathological complexity within ERVK, we implemented an unbiased search for open reading frames in human-derived ERVK provirus sequences. We discovered that ERVK uses frameshifting of the envelope gene transcript to generate a novel protein product with a domain similar to both HIV Tat and marine snail conotoxins, coined ERVK conotoxin-like protein (CTXLP). This observation was alarming, as both these disparate proteins are known to be neurotoxic, in addition to having immunomodulatory effects.

Conotoxins are a type of inhibitor cysteine knot (ICK) proteins and have been previously described as neurotoxins in predatory cone snails (*Conus* sp.) [33]. Conotoxins within the venom of cone snails cause psychosis, paralysis, and fatality in humans [34]. In addition, conotoxin-like protein products of unclear function have been identified in the Nuclear Polyhedrosis Virus (NPV) [35,36,37]. ICK proteins are also called knottins because of their stability, which is imparted by their three disulfide bonds. Two of these disulfide bonds, together with their peptide backbone, form a ring that the third bond goes through, thus forming a “knot” structure [38]. The ICK structure consists of six conserved (connected as CysI-CysIV, CysII-CysV, and CysIII-CysVI) cysteine residues and an otherwise variable peptide backbone [38]. Within the animal kingdom, ICK peptides are found in the venoms of spiders, scorpions, and marine snails, functioning either as pore-blockers or gate-modifiers of ion channels [38]. The classification of knottins is based on criteria such as the gene superfamily, the pattern of cysteine bridging, and molecular targets [33,39]. ERVK CTXLP most resembles O-conotoxins (which encompass ω and γ conotoxin groups); these small toxic peptides act by blocking ion channels [40]. Inhibition of high-voltage activated Ca_V_2.2 (N-type) calcium channels found at the presynaptic terminal of neurons by O-conotoxins, leads to suppressed acetylcholine release and neurotransmission [41]. The success of piscivorous cone snails to envenomate and immobilize their prey by blocking neuromuscular transmission is attributed to the efficacy of conotoxin peptides [34,40].

Given the pathogenic effects of HIV Tat and conotoxins, we hypothesized that ERVK-encoded CTXLP underpins some of the findings associated with transgenic mice expressing the ERVK *env* gene succumbing to motor neuron disease [10]. While the concert of pathological pathways underlying the inception and progression of motor neuron death in ALS is complex, here we focus on the connection between ERVK CTXLP and neuropathological events in ALS, such as inflammation [42], proteinopathy [43], necroptosis [44], and oligodendrocyte perturbation [45,46].

## 2. Materials and Methods

Ethically-sourced human autopsy tissues were obtained from the NIH Neurobiobank and the Veterans Affairs Brain Bank. Cell culture, transfections, cytokine treatments, western blot, chromatin immunoprecipitation, quantitative PCR, immunohistochemistry/histological staining, RNAseq analysis, and computational biology were done as described previously [7,8,47,48,49]. Detailed descriptions of the methodologies are provided in Supplementary Methods.

## 3. Results

### 3.1. An Unbiased Search for Open Reading Frames in the ERVK Genome Identified a Novel env-Derived Protein

**Figure 1:**
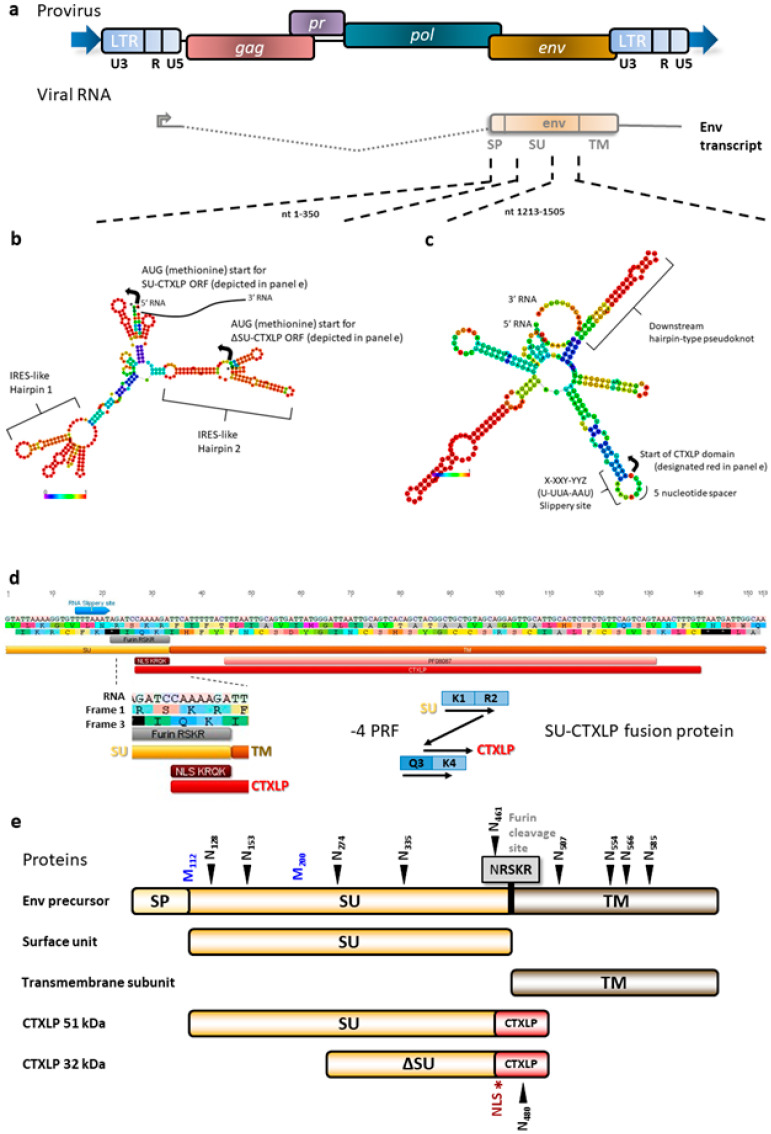
A conotoxin-like protein (CTXLP) is encoded within the endogenous retrovirus-K (ERVK) *env* transcript. (**a**) ERVK proviruses can produce a spliced *env* transcript. ERVK113 was used as a template for subsequent bioinformatic analyses. (**b**) RNAfold analysis predicts two distinct internal ribosomal entry site (IRES)-like hairpins at nucleotides 84–187 and 213–318, potentially allowing for the production of smaller isoforms of ERVK Env surface unit (SU) or CTXLP. (**c**) RNAfold analysis also predicts a conserved -1 ribosomal frameshifting (FS) sequence directly upstream of the CTXLP domain translational start. Three canonical elements i) a slippery site containing an X-XXY-YYZ motif, which after FS by -1, results in XXX-YYY reading, ii) a 5 to 10 nucleotide spacer sequence, and iii) a downstream hairpin-type pseudoknot, may facilitate ribosomal FS into the CTXLP reading frame. (**d**) Ribosomal frameshifting in ERVK *env* can generate a SU-CTXLP fusion protein. A rare -4 FS allows for the translation of the CTXLP cysteine-rich motif at the *C*-terminal end of the SU protein and introduces a nuclear localization sequence (NLS: KRQK motif). (**e**) Diagram of canonical and novel protein products produced by ERVK *env*. The ERVK envelope polyprotein is cleaved by the cellular protease furin (grey, RSKR motif) downstream of the R-X-R/K-R site. This splits the ERVK Env polyprotein into the surface unit (SU) and transmembrane (TM) proteins, which interact to form the viral spike protein on the surface of virions. Post-translational modification of ERVK SU protein includes glycosylation, with N-linked N-X-S/T glycosylation sites identified by black arrows. The first 350 bp of ERVK Env-encoding RNA contains numerous AUG (methionine, blue) translational start sites. Larger (51 kDa) and smaller (32 kDa) CTXLP variants are predicted based on alternative start sites.

A fundamental premise underlying this study was that key mechanisms accounting for the inflammatory pathology associated with ERVK had yet to be discovered. Thus, as an initial line of inquiry, we hypothesized that the ERVK genome encodes more proteins than had been previously described. However, an open reading frame (ORF) analysis of ERVK proviruses yielded no novel conserved domains when a start codon (methionine)-biased analysis was used. Thus, the requirement for an initiating start codon was relaxed. This strategy was appropriate given that alternative ORFs could be accessed through splicing or frameshifting events often employed by retroviruses [50]. The start codon-unbiased analysis delivered a Conserved Domains database hit (Toxin_18, PFAM PF08087), identifying a previously undescribed translation product of the ERVK *env* gene (Figure 1 and Figure S1a). This ORF was in a different reading frame than the *env* gene. It occurred in both type 1 and type 2 ERVK genomes; it was not disrupted by the distinguishing 292-base pair deletion in *env*. Having established the presence of a cryptic ORF, we sought to identify how it might be translated.

Since the reading frames of ERVK *env* (frame +1) and CTXLP (frame +3) differed by -1, ERVK *env* transcripts were examined for evidence of secondary structures that may regulate viral protein production [50], such as internal ribosomal entry sites (IRES) and programmed ribosomal frameshifting (PRF) motifs. Certain viruses, including HIV, use IRES to allow for mRNA translation to begin in the middle of the transcript [51]. These take the form of complex RNA hairpin structures that allow for docking of ribosomal machinery and subsequent protein translation. RNAfold software was used to predict mRNA secondary structure upstream of the CTXLP domain ORF. Within the *env* transcript, we observed RNA secondary structures at nucleotides 84–187 and 213–318, similar to HIV IRES (as predicted by IRESite) that allowed for alternate methionine start codons [52]. Figure 1b depicts that the ERVK *env* transcript contains two typical IRES motifs.

Frameshifting is also a common occurrence in viruses, used to maximize genomic economy and control the ratio of gene products on poly-cistronic transcripts [50,53,54]. Programmed ribosomal frameshifting can occur when three structural RNA elements are combined in the following order: i) a slippery site containing an X-XXY-YYZ motif after which frameshifting by -1 results in an XXX-YYY reading, ii) a five to 10 nucleotide spacer sequence, and iii) a downstream hairpin-type pseudoknot. During the translation of the primary reading frame, the hairpin-type pseudoknot halts the ribosome from continuing translation, leading to ribosomal frameshifting [53]. This configuration allows the downstream hairpin-type pseudoknot to halt the translation of the primary reading frame with the ribosome sitting on the slippery site. The slippery site then re-establishes ribosomal tRNA and mRNA base pairing in the non-primary reading frame and allows for the continuation of translation after the frameshift [53]. This type of ribosomal slippage has been described at the GagPol boundary in HIV [50]. To predict RNA motifs and secondary structure, multiple ERVK *env* nucleotide sequences were submitted to the RNAfold software starting from 150 base pairs upstream of the CTXLP sequence. ERVK CTXLP-encoding transcripts contained an appropriate U-UUA-AAU slippery site followed by a five-nucleotide spacer sequence before the CTXLP domain. All ERVK sequences examined showed a strong probability of forming a hairpin-type pseudoknot within the RNA sequence encoding the CTXLP cysteine-rich motif (Figure 1c). Furthermore, if the envelope protein translates past the CTXLP domain start and frameshifts with a -4 hop, this would introduce a conserved KRQK nuclear localization sequence (NLS [55]) into the hypothetical protein (Figure 1d).

Taken together, this challenges the conventional idea that the ERVK *env* transcript can encode only a single envelope polyprotein, which is proteolytically processed by the cellular enzyme furin into the surface unit (SU) and transmembrane (TM) proteins (Figure 1d) [56]. We predict that programmed ribosomal frameshifting could be used to extend the ORF of the ERVK SU protein by adding on a *C*-terminal CTXLP domain, ultimately creating a novel fusion protein. Moreover, translating *env* from alternative start codons may result in different sized isoforms of CTXLP. Predicted 51 and 32 kDa isoforms of CTXLP likely stem from using the start of the *env* ORF (methionine position 1) or an IRES in the *env* reading frame (starting specifically at methionine position 200), respectively. Our data suggest that the ERVK CTXLP domain is likely expressed as a cryptic peptide through the frameshifted translation of the *env* transcript (Figure 1e).

### 3.2. Characterization of ERVK CTXLP, a Virus-Encoded Conotoxin Protein

**Figure 2:**
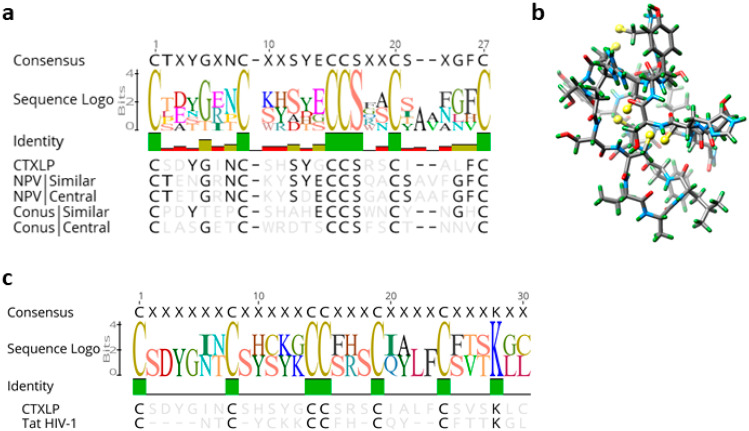
ERVK CTXLP is conserved structurally with *Conus* and viral conotoxins, as well as Human Immunodeficiency Virus (HIV) Tat. (**a**) ERVK CTXLP cysteine-rich motif has a strong similarity to both nuclear polyhedrosis virus (NPV, 46.2%) and *Conus* (45.8%) conotoxin proteins. Cysteine bridges conserved in knottin proteins are indicated with black bars. (**b**) A modeled 3D structure of the CTXLP domain was predicted using the Knotter1D3D software. Note the interactions of the yellow cysteine residues, as they form disulfide bonds. (**c**) Alignment and sequence logo of the cysteine-rich motif in ERVK CTXLP peptide and cysteine-rich domain of HIV-1 Tat protein. Conservation of six of the seven CTXLP cysteine residues is found in HIV Tat, as well as a *C*-terminal lysine residue.

A pBLAST search of the CTXLP domain returns no hits in humans at all, only conotoxin proteins from other species. Given that the ERVK CTXLP domain was predicted to be similar to the Toxin_18 family of proteins known as Conotoxin O-superfamily, we sought to establish the degree of architectural consistency between CTXLP and known conotoxins. The ERVK CTXLP domain is 39 amino acids long, with a core cysteine-rich motif accounting for 30/39 residues (CSDYGINCSHSYGCCSRSCIALFCSVSKLC) (Figure 2a–c and Figure S1b). A sequence logo was generated to assess amino acid conservation between CTXLP and known cysteine-rich proteins (Figure S1b,c, and Table S1). Figure 2a shows the relationship of ERVK CTXLP to representative O-conotoxins and Nuclear Polyhedrosis Virus (NPV) sequences. The ERVK CTXLP domain contained six cysteine residues and one glycine residue, characteristic of the ω-conotoxins, and select γ-conotoxins (Figure S2a) [57]. ERVK CTXLP demonstrated a strong similarity to cone snail O-conotoxins (25.9–45.8% identity) (Figure 2a and Figure S2a). Previous studies have shown that ω-conotoxin’s amino acid residues lysine 2, lysine 4, threonine 11, tyrosine 13, and arginine 22 are important for calcium channel receptor binding [58], of which some residues are similarly conserved in CTXLP (Figure 2a). NPV, which belongs to the insect-infecting Baculoviruses, also produces a conotoxin-like protein (NPV CTXLP) [37]. The ERVK CTXLP domain showed the greatest similarity to NPV viral proteins (max similarity 46.2%, Figure 2a and Figure S2a–c). Conotoxins adopt a knot-like protein conformation, called a knottin structure, which is important for their stability and action. O-conotoxin and NPV CTXLP knottins include three disulfide bonds, formed through cysteine bridges (Figure S1b). Apart from the cysteine motif, amino acid residues in conotoxins are highly variable [57], suggesting that the conservation of the cysteine residues and, therefore, the tertiary structure is more important for peptide function than the primary amino acid sequence. Tertiary structure prediction of the ERVK113 CTXLP protein using Knotter 1D3D software resulted in the predicted 3-dimensional structure shown in Figure 2b, highlighting its capacity to form appropriate cysteine bridges as found in knottin proteins. Thus, we conclude that an ERVK SU-CTXLP fusion protein is likely to harbor a *C*-terminal knottin globular domain, based on sequence similarity with other conotoxin proteins and conservation of the core cysteine motif.

After identifying that two unrelated groups of viruses (ERVK and NPVs) both have conotoxin-like protein-coding capacity, we also searched for conotoxin-like peptides within translations of all three forward reading frames of the *env* region of several other retroviral genomes (HIV-1, HTLV-1, MMTV, ERVW, ERVH; Table S1). No conotoxin-like domains were identified using HMMER and BLAST searches in any of these retroviruses, indicating that the CTXLP signature may be a unique feature of ERVK proviruses. However, comparative protein analyses revealed a similarity between the CTXLP cysteine motif and the retroviral accessory protein HIV Tat (Figure 2c). When ERVK CTXLP was aligned to the cysteine-rich region (residues 22–44) of Tat proteins from HIV-1, some degree of similarity was detected (23.3% identity). The conserved C-C-CC-C-C motif of Tat has a tighter cysteine spacing than ERVK CTXLP, or conotoxins. Tat toxicity and its ability to regulate gene expression centers around the use of its cysteine-rich domain [59,60]. In driving a self-promoting inflammatory environment for HIV, Tat upregulation of NF-κB relies on its cysteine-rich motif [60,61]. These data suggest that HIV Tat and ERVK CTXLP may share some functional similarities.

### 3.3. ERVK CTXLP Variants in the Human Genome and Primate Homologs

A tBLASTn search of all human sequences in the NCBI reference database (nr) with the CTXLP domain of ERVK-10 returns only known ERVK loci (e-value < 1). Within the human reference and alternative genome builds, there are at least 14 ERVK insertions (including ERVK-1, -18, -20, -24, -111, -113, and -115) capable of encoding a full-length SU-CTXLP fusion protein. Analysis of ERVK CTXLP domain variants revealed a high degree of sequence conservation, with a single common allele dominating (Figure S3a). Additionally, several CTXLP-encoding ERVK proviruses have been associated with human disease states (Table S2), but none of the identified CTXLP domain polymorphisms correlated with disease. While CTXLP-containing ERVK proviruses can also be found in other primates (Figure S3b–d), the predominant form of ERVK CTXLP in humans (from ERVK-113) was examined further as the prototypic model for this viral peptide. Moreover, this CTXLP signature can be found in a murine model of ERVK *env*-driven ALS-like neuropathology (Figure S3e) [10].

### 3.4. Novel Inflammation-Inducible ERVK CTXLP Protein is Distinct from Other ERVK Gene Products

The characterization of ERVK CTXLP is paramount to our understanding of ERVK in health and disease. Therefore, we developed ERVK CTXLP specific reagents to study this novel viral protein. A custom rabbit antibody was generated and validated using i) competitive peptide blocking assay (Figure S4a), ii) immunoprecipitation and western blot for CTXLP and surface unit (SU) epitopes (Figure S4b), iii) comparison between pre-immune serum and post-CTXLP peptide immunization antibodies in immunohistochemistry (Figure S4c), and iv) overexpression of ERVK CTXLP and SU pcDNA3.1 vectors (Figure S4d). These multiple approaches support the specificity and use of these reagents in this study.

### 3.5. ERVK CTXLP Is Inducible through the Action of Pro-Inflammatory Signaling

**Figure 3:**
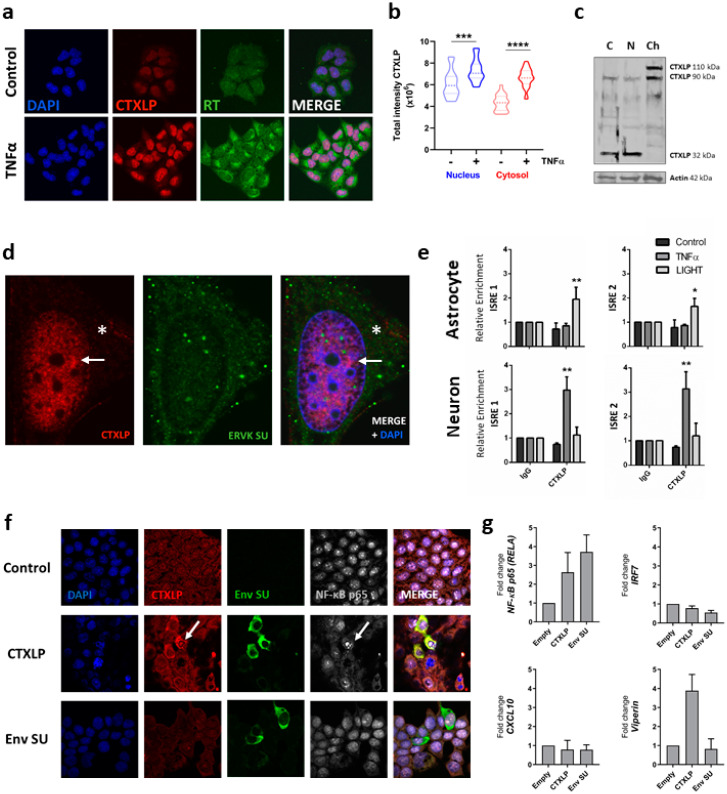
ERVK CTXLP is induced by inflammatory signals. (**a**) The pro-inflammatory cytokine Tumor Necrosis Factor-alpha (TNFα) enhances ERVK CTXLP levels in human astrocytic SVGA cell line. Representative confocal micrographs depicting ERVK CTXLP (red) and ERVK reverse transcriptase (RT, green) in cells treated with or without 0.1 ng/mL TNFα for 24 h, *n* = 2. DAPI stain indicates nuclei (blue). CTXLP mainly localized to the nucleus with diffuse cytoplasmic staining. (**b**) Quantification of CTXLP staining in untreated and TNFα-treated SVGA cells highlights the increase in both nuclear and cytoplasmic expression of CTXLP under inflammatory conditions (*** *p* < 0.001, **** *p* < 0.0001). (**c**) Cell fractionation further supported the nuclear localization of CTXLP proteins in SVGA cells. Cytoplasmic (C) and nuclear fractions (N) expressed mainly the small form of CTXLP (32 kDa), whereas larger (90–110 kDa) isoforms of CTXLP were mostly confined within the cellular chromatin (CHR) fraction (*n* = 4). (**d**) Upon TNFα treatment, although the expression of CTXLP and Env SU proteins was enhanced in SVGAs, they did not co-localize upon merging confocal microscopy images. CTXLP nuclear foci are indicated by an arrow, whereas cytoplasmic puncta are indicated by an asterisk. Together, this reveals that CTXLP expression exhibits a distinct cellular distribution pattern from that of SU. (**e**) CTXLP protein binds chromatin, and specifically at regions containing interferon-stimulated response elements (ISREs) and κB sites within the ERVK promoter (5′ LTR) [62]. Chromatin immunoprecipitation (ChIP) was performed following 8 h of 10 ng/mL TNFα or LIGHT (cytokine belonging to the TNF superfamily) treatment in SVGA cells (*n* = 3) and human ReNcell-derived neurons (*n* = 2). There was a notable increase in CTXLP chromatin binding in astrocytes and neurons upon stimulation with LIGHT and TNFα, respectively (* *p* < 0.05, ** *p* < 0.01). (**f**) 293T cells were transfected with pcDNA3.1 plasmids encoding empty vector, ERVK CTXLP, or ERVK SU for 24 h. Cells were assessed for expression of CTXLP or Env SU epitopes using a custom CTXLP antibody or a commercial ERVK Env SU antibody. Note that overexpression of the ERVK Env SU construct only generates an overexpressed protein containing the SU epitope, whereas CTXLP overexpression generates an overexpressed fusion protein containing both ERVK Env SU and the cysteine-rich CTXLP domain epitopes. Only CTXLP overexpression results in NF-κB p65 nuclear translocation (arrow), indicative of NF-κB activation. DAPI stain depicts nuclei. (**g**) Q-PCR analysis of inflammatory genes in 293T cells transfected with pcDNA3.1 plasmids encoding empty vector, ERVK CTXLP, or ERVK SU for 24 h (*n* = 2). Note the enhanced expression of NF-κB p65 transcript in both CTXLP and SU transfected cells. Conversely, only CTXLP-transfected cells exhibit enhanced Viperin mRNA expression. No significant change in IRF7 or CXCL10 transcript abundance was observed.

Pro-inflammatory cytokines have been shown to induce ERVK expression [8,47,62]. Upon Tumor Necrosis Factor-alpha (TNFα) treatment, enhancement of ERVK CTXLP expression was interrelated with cytoplasmic ERVK reverse transcriptase (RT) levels in astrocytic cell lines (Figure 3a). This is consistent with previously observed global regulation of ERVK gene expression by pro-inflammatory stimuli [8]. Endogenous CTXLP protein was located predominantly in the nucleus of SVGA cells, as seen in both confocal imaging (Figure 3a,b) and a chromatin cellular fraction blot (Figure 3c). Higher resolution images and quantification of TNFα-treated CTXLP expressing astrocytes showed that CTXLP puncta also formed in the cytoplasm, suggesting that potential isoforms of CTXLP may have location-specific functions (Figure 3c,d). ERVK CTXLP expression did not co-localize with the canonical ERVK SU protein (as measured with a commercial antibody), indicating that these proteins have different localization sequences (i.e., NLS in CTXLP, and not SU) and cellular distribution patterns. Despite the presence of SU epitopes in denatured CTXLP peptides (Figure S4b), the in vivo protein conformation may mask these epitopes [63], leading to the appearance of distinct cellular distributions for these ERVK proteins.

### 3.6. ERVK CTXLP Binds Chromatin

Consistent with our observation that CTXLP is enriched in the chromatin fraction (Figure 3c), DNABIND predicts that CTXLP binds DNA, with a score of 1.771 and a probability of DNA binding of 85.5%, which are firmly above threshold cut-offs [64]. As HIV Tat is known to regulate viral and cellular gene expression through the modulation of NF-κB activity and interaction with κB sites [61,65], we sought to determine if CTXLP has similar properties. We have previously published that the ERVK promoter contains two interferon-stimulated response elements (ISREs) that overlap with adjacent κB sites for interaction with NF-κB transcription factors [8,62]. Treatment of human astrocytic and neuronal cell lines with pro-inflammatory cytokines belonging to the TNF superfamily, TNFα and LIGHT (lymphotoxin-like inducible protein that competes with glycoprotein D for herpes virus entry on T cells), enhances NF-kB binding to these sites [8]. Experimentally, chromatin immunoprecipitation revealed that CTXLP bound the ISREs within the ERVK viral promoter (5′ LTR) (Figure 3e). Notably, enhanced chromatin association of CTXLP occurs in the presence of inflammatory cytokine stimuli, although the provoking inflammatory stimuli can vary between cell types. Together with cell fractionation data, this suggests that CTXLP binds DNA and, therefore, might regulate gene expression. Indeed, ectopic expression of CTXLP (but not ERVK SU) enhanced nuclear NF-κB p65 expression (Figure 3f,g), as does HIV Tat [61]. We also assessed the expression of other ISRE and κB regulated inflammatory genes that may be impacted by ERVK CTXLP. The mRNA expression of the transcription factor IRF7 or chemokine CXCL10 was not impacted by the presence of ERVK CTXLP or SU (Figure 3g). However, ERVK CTXLP (but not ERVK SU) enhanced viperin transcript expression (Figure 3g), suggesting potential impacts on metabolism and immunity [66]. These data indicate that ERVK CTXLP can modulate cellular gene expression.

Several cell types are known to produce ERVK *env*-derived proteins and even form virions in certain disease states, notably in various types of cancers (reviewed in [30]). Likewise, the teratocarcinoma NCCIT and the breast cancer T47D cell lines express ERVK at high levels [67,68]. To assess the extent and localization of CTXLP expression in such ERVK^+^ cells, we screened a panel of cancer cell lines (Figure S5). Notably, CTXLP was not restricted to the nucleus in some cancerous cells. In the NCCIT cancer cell line, endogenous CTXLP protein appeared ubiquitously expressed and localized to the cytoplasmic, nuclear and chromatin enriched fractions (Figure S5a,b). CTXLP protein was also found in both soluble and insoluble protein preparations from NCCIT cells. The latter suggests that there is an interaction of CTXLP with cell membranes and chromatin. The abundant and ubiquitous nature of CTXLP expression in untreated NCCIT cells, as compared with untreated human astrocytes (SVGA cells), was further supported by confocal imaging (Figure S5a). Moreover, several other cancer cell lines express CTXLP (Figure S5a,c), including the T47D breast cancer cell line, which has been previously associated with ERVK reactivation [68]. These findings underscore the fact that ERVK CTXLP may localize to distinct cellular compartments depending on the cell type and cellular state.

### 3.7. ERVK CTXLP Protein Is Associated with Pathological Features of Amyotrophic Lateral Sclerosis (ALS)

The hallmark pathology of ALS involves motor neuron damage within the motor cortex and spinal cord. Degeneration of motor neurons in Brodmann’s areas BA4 and BA6 (the primary motor and pre-motor cortex areas, respectively), as well as axonal disruption in the spinal cord, leads to denervation culminating in muscle atrophy. Therefore, we examined ex vivo (autopsy) human tissues for the expression of ERVK CTXLP in conjunction with pathological markers indicative of cell death. Despite cell line models indicating that CTXLP can induce apoptosis (Figure S4d–f), in preliminary immunopathological studies, we did not observe substantial caspase-3 staining in ALS tissues. Alternatively, necroptosis is a form of cell death that drives inflammation and is posited to contribute to ALS neuropathology [69,70]. Activation of this pathway involves RIP1/RIP3 signaling leading to phosphorylation and multimerization of the effector protein mixed lineage kinase domain-like (MLKL), which forms pores in the plasma membrane; this allows the release of cellular contents into the extracellular space and drives a local inflammatory response [44]. The cellular redistribution of cytoplasmic MLKL protein to the plasma membrane is characteristic of ongoing necroptosis [71]. To our knowledge, this is the first report to quantitatively assess MLKL expression patterns in disease-affected tissue from patients with ALS.

### 3.8. CTXLP Is Associated with Neuronal Necroptosis in ALS

**Figure 4:**
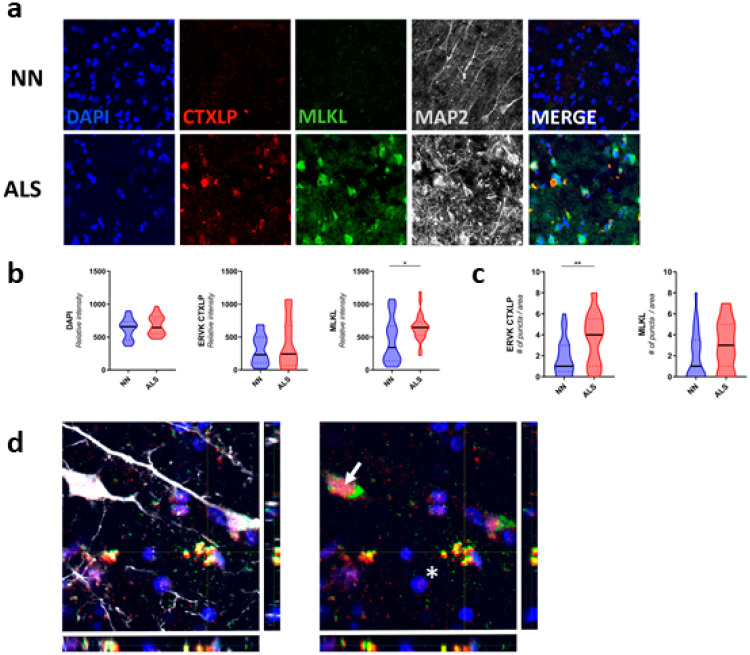
ERVK CTXLP is enhanced in the motor cortex of patients with Amyotrophic Lateral Sclerosis (ALS) and associated with necroptosis. (**a**) ERVK CTXLP levels are enhanced in motor cortex brain tissues of patients with ALS, as measured by confocal microscopy. Representative 40× confocal micrographs of ERVK CTXLP (red), mixed lineage kinase domain-like (MLKL) protein (necroptosis marker, green) and neuronal microtubule-associated protein 2 (MAP2) expression (grey) in Brodmann area 6 (BA6, panel D) pre-motor cortex tissue of a neuro-normal (NN) control (*n* = 5) and patient with ALS (*n* = 5). DAPI stain depicts nuclei. (**b**) Violin plots of staining quantification of DAPI, CTXLP, and MLKL in NN and ALS cohorts for BA6 tissue. (**c**) Violin plots of protein puncta quantification of CTXLP and MLKL in NN and ALS cohorts for BA6 tissue). (**d**) Enhanced expression of MLKL in CTXLP^+^ neurons from the motor cortex of a patient with ALS (arrow). The nearby degenerating cell is marked with co-localized CTXLP^+^MLKL^+^ puncta (asterisk). Statistical test with unpaired two-tailed *t*-tests, (* *p* < 0.05 ** *p* < 0.01, black bars are medians).

**Figure 5:**
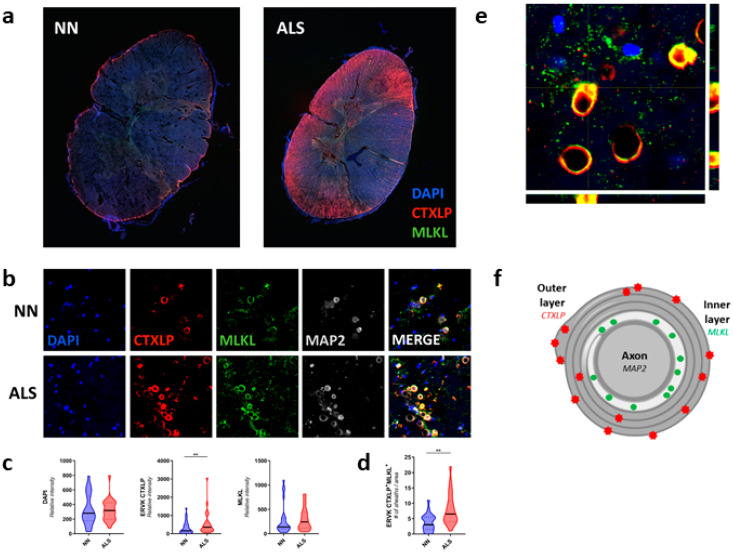
ERVK CTXLP is enhanced in the spinal cord of patients with ALS and associated with axonal sheaths. (**a**) ERVK CTXLP levels are enhanced in autopsy spinal cord tissues of patients with ALS, as measured by confocal microscopy. Representative 10× mosaic confocal micrographs of ERVK CTXLP (red), MLKL (necroptosis marker, green), and DAPI (nuclear marker, blue) in the cervical spinal cord of a neuro-normal (NN) control and a patient with ALS. Note the intense CTXLP staining in the lateral corticospinal tract, surrounding a lesioned area in ALS. (**b**) Representative 40× confocal micrographs of ERVK CTXLP (red), MLKL (necroptosis marker, green), and neuronal MAP2 expression (grey) in the cervical spinal cord of a NN control (*n* = 5) and a patient with ALS (*n* = 5). DAPI stain depicts nuclei. (**c**) Violin plots of staining quantification of DAPI, CTXLP, and MLKL in NN and ALS cohorts for cervical spinal cord. (**d**) Violin plots of protein quantification of CTXLP^+^MLKL^+^ sheaths in cervical spinal cord. Statistical test with unpaired two-tailed *t*-tests (** *p* < 0.01, black bars are medians). (**e**) Projected image of a 5 μm tissue section depicting CTXLP^+^MLKL^+^ sheaths in the cervical spinal cord of a patient with ALS. The oligodendrocyte cell body is indicated by an asterisk and myelin sheath by an arrow. CTXLP^+^ rings ranged from 6–16 μM in diameter, with clear differences in the thickness of the sheaths surrounding axons. (**f**) Diagram of CTXLP^+^MLKL^+^ sheaths surrounding neuronal axons in ALS. Image produced with BioRender and PowerPoint.

Confocal microscopy of the motor cortex (Figure 4) and spinal cord (Figure 5) specimens from neuro-normal controls and patients with ALS revealed substantially enhanced CTXLP protein expression in ALS (BA4, *p* < 0.01 (Figure S6), BA6, NS (Figure 4)). This viral protein may be derived from select ERVK loci encoding the CTXLP domain, which are more abundantly expressed in ALS cases than controls (Figure S7a). In the motor cortex, CTXLP^+^ cells were predominantly neurons (based on MAP2 neuronal marker) (Figure 4a, Figure S6a and Figure S7b), although ERVK CTXLP^+^MAP2^−^ cells with astrocytic morphology were observed in select ALS cases (Figure S7c). This is consistent with previous observations of ERVK protein production in the motor cortex of patients with ALS [6,7,8]. Notably, basal CTXLP expression was mostly nuclear in neuro-normal tissues, whereas CTXLP exhibited a pattern of cytoplasmic aggregation in motor cortex tissues from patients with ALS (puncta count for BA4, *p* < 0.0001, BA6, *p* < 0.01) (Figure 4a,c,d, Figure S6a,c and Figure S7b). ERVK CTXLP expression was also strongly associated with elevated MLKL levels in ALS as compared to neuro-normal controls (BA4, NS trend (Figure S6b,c), BA6, *p* < 0.05 (Figure 4b,c)). As with CTXLP expression in ALS, MLKL redistribution could be observed in intact cells, as well as degenerating cells. Figure 4d shows an example of an ALS tissue containing a CTXLP^+^ neuron with an accumulation of MLKL staining in the axon hillock (arrow), as well as a nearby degenerating cell (co-localized CTXLP^+^MLKL^+^ puncta, asterisk). Typical of necroptosis [72], extracellular CTXLP deposits were also observed surrounding degenerating pyramidal neurons in the motor cortex of ALS patients (Figure 4d and Figure S7b). Together, these pathological indicators suggest CTXLP-driven proteinopathy, neuronal damage, and necroptosis, causing the release of CTXLP in the motor cortex of patients with ALS.

### 3.9. CTXLP Is Associated with Oligodendrocyte Perturbation in ALS

Apart from motor neuron disturbances, accumulating evidence points to oligodendrocyte (OL) dysfunction and demyelination in ALS (reviewed in [73]). Specifically, the anterior and lateral corticospinal tracts are impacted in ALS [74]. Figure 5a points to the dramatic CTXLP staining in spinal cords from patients with ALS versus neuro-normal controls, with distinctive expression in the lateral corticospinal tract (*p* < 0.01, Figure 5c). Remarkably, CTXLP patterning in the spinal cord exhibited a ring pattern surrounding MAP2^+^ neurons (MAP2 marks neuronal axons in grey, Figure 5b). This distinctive staining pattern pointed towards CTXLP expression in OLs, as these cells wrap protective myelin sheaths around axons in the spinal cord [75]. Many of these sheaths were also MLKL positive, with a significant increase in numbers of CTXLP^+^MLKL^+^ cells in ALS as compared to controls (*p* < 0.01, Figure 5d). This is notable, as Ser441 phosphorylation of MLKL is associated with myelin destabilization following tissue injury [76]. A projection of ALS tissue staining reveals a consistent pattern of co-localized CTXLP and MLKL, with additional CTXLP staining beyond the OL cell body (asterisk) and into the tight wrappings of the myelin sheath surrounding neuronal axons (arrow) (Figure 5e,f).

**Figure 6:**
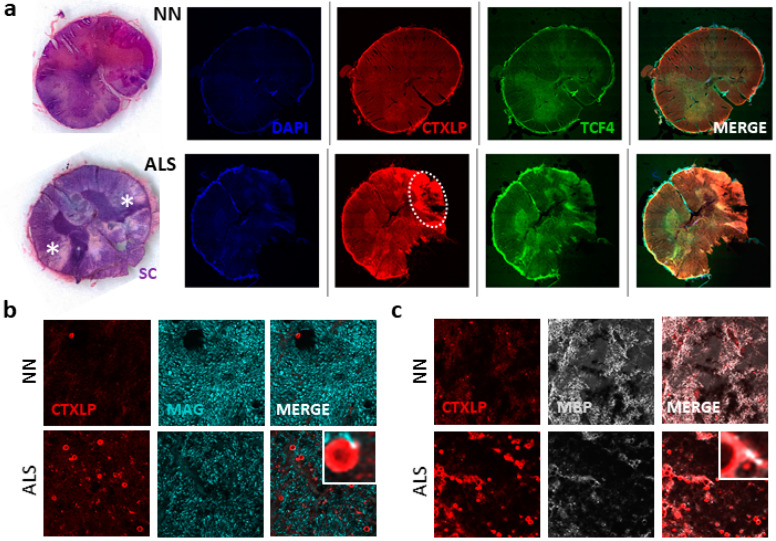
ERVK CTXLP protein expression is associated with demyelinated lesions in spinal cord tissues from patients with ALS. (**a**) ERVK CTXLP levels are enhanced in autopsy lumbar spinal cord tissues from patients with ALS, as measured by light and confocal microscopy. Representative 10× confocal micrographs of ERVK CTXLP expression in ex vivo lumbar (LC) spinal cord of a neuro-normal control (NN, *n* = 5), and patients with ALS (*n* = 5). Solochrome cyanine (SC) stain (purple) with eosin counterstain (pink) depicts tissue myelination; pale lesions appear in ALS tissues (as indicated by an asterisk). These lesioned areas exhibited increased CTXLP protein expression (red). Oligodendrocyte precursor marker transcription factor 4 (TCF4) is in green. DAPI stain depicts cellular nuclei. Note: CTXLP expression occurs in either the lateral (indicated by a white circle) and anterior cortical spinal tracts. (**b**) Myelin protein MAG levels are lower in CTXLP^+^ ALS-lesioned spinal cord tissues (*n* = 5), as compared to controls (*n* = 5). (**c**) Myelin protein MBP levels are reduced in CTXLP^+^ ALS lesioned spinal cord tissues (*n* = 5), as compared to controls (*n* = 5). The magnified inset depicts MBP-degraded myelin vesicles (condensed MBP aggregates) in CTXLP-expressing cells, which is indicative of an ongoing neurodegenerative process.

Next, we investigated the association of ERVK CTXLP expression and myelin damage in ALS. Our observations showed that CTXLP expression occurs in either lateral and anterior cortical spinal tracts in ALS (Figure 6a and Figure S8). Strong CTXLP^+^ staining coincides with demyelinating lesions, as shown by solochrome cyanine staining of adjacent tissues (Figure 6a and Figure S8b). Additionally, the overall levels of key mature OL proteins, myelin-associated glycoprotein (MAG), and myelin basic protein (MBP) were greatly diminished in cervical spinal cords from patients with ALS (Figure 6b,c). Evidence of MBP-forming degraded myelin vesicles (Figure 6c inset) was also apparent in CTXLP^+^ OLs, further indicating an underlying neurodegenerative process [77].

**Figure 7:**
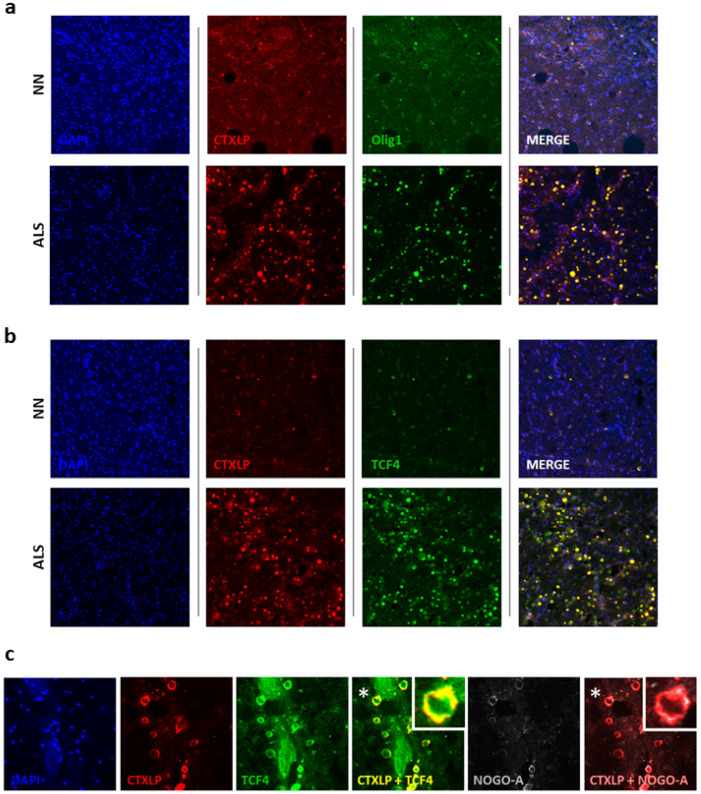
Nogo-A expression in CTXLP^+^ oligodendrocyte precursors may limit spinal cord remyelination in ALS. ERVK CTXLP levels are associated with demyelination, and CTXLP is enhanced in TCF4+Olig1+ oligodendrocyte precursors in cervical spinal cord tissues from patients with ALS. (**a**,**b**) ERVK CTXLP expression in ex vivo cervical (CC) spinal cord of NN controls (*n* = 5) and patients with ALS (*n* = 5) analyzed by confocal imaging (representative 20× confocal micrographs are shown). In ALS tissue, CTXLP expression (red) co-localizes with Olig1 (green, panel A) or TCF4 (green, panel B) markers indicative of oligodendrocyte precursor cells. DAPI stain depicts cellular nuclei. (**c**) ERVK CTXLP^+^ oligodendrocyte precursors either express myelin inhibitory protein Nogo-A or lie in close proximity to Nogo-A positive cells (Figure S9b) in spinal cord tissues of patients with ALS. Human ex vivo cervical spinal cord tissues were stained for ERVK CTXLP (red), TCF4 (green), Nogo-A (grey), and nuclei (blue) in NN controls (*n* = 5) and patients with ALS (*n* = 5). Image merging for CTXLP and TCF4 indicates that oligodendrocyte precursors express CTXLP in ALS. White stars indicate areas that are magnified to depict overlapping protein expression in CTXLP^+^ rings.

Further evidence indicates that ERVK CTXLP is associated with altered OL behavior. CTXLP expression in the spinal cord of patients with ALS was strongly associated with increased transcription factor 4 (TCF4, oligodendrocyte precursor marker) expression (Figure 7). Oligodendrocyte precursor cells (OPCs) are a pool of immature OLs, which express characteristic markers such as TCF4, oligodendrocyte transcription factor 1 (Olig1), and oligodendrocyte transcription factor 2 (Olig2) [78]. Upon differentiation into mature OLs, they begin to express myelin proteins such as proteolipid protein (PLP), myelin basic protein (MBP), myelin oligodendrocyte glycoprotein (MOG), and myelin-associated glycoprotein (MAG) [78]. Oligodendrocytes must myelinate early post-differentiation, and myelination occurs within a short timeframe (12–18 h), where their extended processes ensheathe 50–60 axonal segments simultaneously [79]. Pools of OPCs can remain in tissues and are capable of migration and later differentiation into mature OLs, often in response to brain injury [80]. However, in many disease states, an attempt at remyelination is most often unsuccessful [80]. A prevailing theory surrounding defects in remyelination is that despite increased numbers of OPCs in injured tissue, these precursor cells become stalled in an immature state and fail to differentiate into mature OLs properly [80]. Alterations in OPC markers, such as enhanced levels of TCF4 and Olig1 occurs in tissue lesions from patients with MS [81]. Figure 7a,b depicts an enhanced number of CTXLP^+^ OPCs in ALS. Colocalization of TCF4 and Olig1 with CTXLP expression in the spinal cord of patients with ALS indicates that these cells are indeed immature OLs.

Neurite outgrowth inhibitor (Nogo-A) is a key regulator of OPC differentiation; when OPCs express Nogo-A they are unable to progress towards a mature OL phenotype capable of myelination [82,83]. Thus, enhanced expression of Nogo-A in OPCs in the context of inflammation and disease states prevents axonal regeneration by restricting OPC maturation [84]. As an example, demyelinated MS lesions show an increased abundance of Nogo-A^+^ OPCs, and the inability of OPCs to mature is proposed as the mechanism driving a non-permissive environment leading to remyelination failure [84,85]. Western blot analysis confirmed that Nogo-A expression was substantially elevated in ALS spinal cord (Figure S9a; *p* < 0.05). Figure 7c demonstrates that CTXLP expression in the spinal cord of patients with ALS is associated with elevated Nogo-A expression, particularly in OPCs (patient 1, Figure 7c) and other cell types (patient 2, Figure S9b). This specifically occurs in areas of myelin depletion (see Figure 6b,c). It has been demonstrated in the human spinal cord that select myelin protein rings (PLP, MOG, but not MAG) are detectable by immunohistochemistry even three years after injury in degenerating fiber tracts exhibiting the absence of intact axons [86]. Therefore, degrading CTXLP^+^MLKL^+^Nogo-A^+^ myelin rings may persist in patients with ALS for most of the progressive phase of the disease (2–5 years) [74]. Together, CTXLP toxicity, MLKL effects, and Nogo-A expression in degenerating ALS tissues may ultimately create a non-permissive environment for neural regeneration [86], contributing to the rapid clinical progression of this neurodegenerative disease in select ALS patients.

## 4. Discussion

A pathological role for ERVK *env* in ALS is currently contentious [10,11,13]. Transgenic mice with neuron-restricted ERVK *env* expression, as well as in vitro models, support the toxic potential of the ERVK *env* gene [10,87,88,89]. In these systems, toxicity has been attributed to either the SU or TM proteins. Recently, Mayer et al. have proposed that proteins other than canonical full-length Env should be considered when examining the role of ERVK in ALS disease pathology [11]. Herein, we have described a novel protein product termed conotoxin-like protein (CTXLP), which is cryptically encoded within ERVK *env* and may further elucidate the toxic nature of ERVK reactivation in the motor cortex and spinal cord. ERVK CTXLP spans several pathological features, as it encompasses properties of an ALS risk gene, viral protein, and toxin.

Neurotoxins have long been proposed as etiological agents of ALS. The most prominent example is the suspected link between ALS (or an ALS-like syndrome) and beta-*N*-methylamino-l-alanine (BMAA), a neurotoxin produced by a group of terrestrial cyanobacterial symbionts in cycad plants [90]. However, large scale spatial clustering of individuals with ALS has been inconsistent with the range of BMAA-producing cyanobacteria and other suspected environmental risk factors [90]. That said, no proposed neurotoxin-based etiology has been able to explain the predominance of sporadic cases of ALS. Therefore, although an environmental neurotoxin model for ALS makes sense at a physiological level, a genetic-based model (with environmental/epigenetic influence) seems more likely at an epidemiological level. A genetically encoded neurotoxin such as ERVK CTXLP would be consistent with both theories. Environmental triggers which promote NF-κB signaling in the context of immunity-related risk genes for ALS/FTD [91,92,93], may lead to a failure to control the expression of this endogenous viral symbiont, thus facilitating ERVK CTXLP-driven neuropathology.

Our work suggests that the enhancement of CTXLP and its putative toxic effects are likely driven in part by NF-κB signaling. In the context of TNFα-related inflammatory signaling, CTXLP bound ISRE and κB sites in the ERVK promoter (Figure 3e). Enhanced expression of CTXLP further augmented NF-κB p65 transcript levels and nuclear localization (Figure 3f,g). Thus, NF-κB driven expression of a human-genome encoded viral protein like CTXLP could mimic a gene-environment interaction and begin to explain how ERVK may pathologically contribute to a subset of sporadic cases of ALS.

It has previously been observed, in a seminal study by Li et al., that patients with ALS express ERVK Env surface unit protein in their frontal cortex and anterior horn motor neurons in the lumbar spinal cord [10]. Approximately half of the patients with ALS in this study (11/19) expressed CTXLP at levels above controls in the motor cortex and spinal cord (specifically the lateral and anterior horns), further supporting a role for ERVK-driven pathology in at least a subset of ALS patients [7,8,10]. It may be that ERVK CTXLP effects were previously implicated in ALS pathology. The most obvious case for this claim is the phenotype of transgenic mice with neuron-restricted expression of ERVK *env*, which develop progressive motor pathology and symptoms, causing a 50% case fatality within 10 months [10]. An analysis of the *env* gene insert in these transgenic mice reveals that they have the capacity to produce CTXLP (Figure S3e), and that neuronal production of this novel viral protein may have been occurring in this model system. It will be a serious future undertaking to decipher the relative contributions of canonical *env* proteins versus CTXLP in these transgenic mice and other systems. Moreover, the use of an SU-CTXLP fusion protein may have helped the exogenous form of ERVK overcome the retrovirus’ natural tropism with only SU. Peptide addition to retroviral SU proteins can target virions to other cell types and impact SU glycosylation patterns [94,95]. In addition to the many roles of env-derived proteins [30], the SU domain may facilitate the secretion of CTXLP; future research in this area is warranted.

The importance of the identification of CTXLP protein expression patterns in ALS is upheld by the lack of correlation with total ERVK *env* transcript abundance. Consistent with a recent study by Mayer et al. [11], we observed a lack of differential total ERVK *env* RNA expression in controls versus the ALS cohort (Figure S7a). However, PCA analysis revealed that the expression of select ERVK CTXLP^+^ loci cluster the ALS cohort versus controls, suggesting that specific CTXLP loci may drive the expression of CTXLP protein in ALS. There are notable alterations in RNA translation in ALS proteinopathy [96,97]; thus, deregulated processes may favor CTXLP expression over canonical ERVK *env* protein products. Our data from protein analysis shows obvious differences in CTXLP expression between clinical groups (Figure 4, Figure 5, Figure 6 and Figure 7). Moreover, ERVK CTXLP expression was primarily observed in ALS disease-affected tissues, specifically in the primary motor cortex (BA4) and the lateral corticospinal tract. Cryptic peptides, such as CTXLP, often have significantly different functions than their precursor proteins [98]. Indeed, we observe different cellular localization of the ERVK SU protein as compared to ERVK CTXLP. In cell line models and neuro-normal tissues, basal expression of ERVK CTXLP was mainly nuclear; however, in diseased tissue, there was evidence of CTXLP protein deposits in the cytoplasm of neurons within the motor cortex and myelin sheaths within the spinal cord. Our neuropathological observations point to possible roles for ERVK CTXLP in necroptosis, expansion of OPCs, and demyelination.

The role of necroptosis in ALS remains controversial, as motor neuron disease in murine models is independent of necroptosis signaling and MLKL activation [99,100]. In contrast, recent in vitro work using a fully-humanized co-culture system demonstrated that an unidentified toxic factor secreted from primary astrocytes from sporadic ALS, but not those from control patients, triggered necroptosis-mediated death of motor neurons [69]. We postulate that ERVK CTXLP could play a role in inflammatory cell death, based on our observations of enhanced aggregates of MLKL in CTXLP^+^ neurons in ALS brain tissue. Enhanced MLKL expression was also observed in CTXLP^+^ OPCs in the spinal tissue from patients with ALS, which may not causally link to necroptosis per se, but with myelin breakdown and regeneration [76]. Our work supports the accumulating evidence of OL pathology in ALS [101] and points to CTXLP as also having a potential role in OL pathology in a select subset of ALS cases.

CTXLP expression was also tightly correlated with Nogo-A levels in the spinal cord of patients with ALS. Nogo-A has been previously identified as a prognostic marker and therapeutic target in ALS due to its substantial expression in motor neuron disease and destabilizing effect on neuromuscular junctions [102,103]. As expected [104,105], elevated Nogo-A expression was associated with evidence of spinal cord injury and increased OPC numbers in this study. In ALS tissues, CTXLP expression was observed in conjunction with elevated Nogo-A in the sheaths, which is expected to limit neurite outgrowth and prevent myelination, coinciding with reduced MAG and MBP expression observed in ALS tissues. The blockade of voltage gated calcium channels (VGCCs) induces the expression of Nogo-A [105]; given the similarity of CTXLP with O-conotoxins which are known to block select VGCCs [33], this may be a mechanism of action in ALS that ought to be explored. Taken together, the impact of CTXLP, MLKL, and Nogo-A on OPC maturation and myelination likely results in unprotected and susceptible axons in ALS-lesioned areas of the corticospinal tracts. Our study implicates a novel toxic ERVK protein in MLKL-driven pathology and myelin damage in ALS neurodegeneration and broadens the outlook on potential therapeutic strategies for ALS.

We envision that a substantial amount of research is required to address potential mechanisms of ERVK CTXLP neuropathology, as ERVK CTXLP is a putative functional homolog of HIV Tat. The mechanisms surrounding HIV Tat neurotoxicity are diverse and manifold [106,107]. The cysteine-rich motif of Tat endows this protein with neurotoxic properties [108]. Tat expression in the brains of HIV-1 infected patients has been associated with neuronal and oligodendrocyte apoptosis via caspase activation and calcium accumulation [107,109]. The cysteine motif in HIV Tat has also been associated with increased HIV transactivation and global gene regulation by interacting with transcriptional machinery [110,111]. HIV Tat can also transactivate ERVK [112]. Thus, the multiple functions of HIV Tat suggest that CTXLP’s similar cysteine motif may also contribute to neurotoxicity and gene regulation. There are no known small molecule drugs to inhibit the action of conotoxins, yet there is ongoing development on anti-Tat therapeutics [113]. Considering the structural and functional similarities between HIV Tat and ERVK CTXLP, small molecule inhibitors may serve as a therapeutic approach for motor neuron disease. Research in these areas is currently underway.

Revisiting our understanding of ERVK has shed new light on this endogenous viral symbiont. Here we have shown that a ribosomal frameshifting event in the ERVK *env* transcript allows for the formation of novel fusion protein we have called ERVK CTXLP. Our findings reveal an unforeseen complexity within the ERVK genome and highlight a biological curiosity in that a virus-encoded neurotoxin is hiding within the human genome. CTXLP is strongly upregulated by inflammatory NF-κB signaling. As ERVK CTXLP is present in affected CNS tissues of many ALS patients, it may be a useful biomarker for the disease, following independent confirmation of this initial study. Furthermore, as a putative etiological agent of ALS (and other ERVK-associated diseases [23]), the discovery of ERVK CTXLP is likely to have implications beyond ALS neuropathology and therapeutics, as it speaks to a broader challenge in accurately assessing the role of retroelements in health and disease. Fundamentally, these data further support the concept that human cells are built using a holobiontic template of human and viral genes, blurring the lines between “human” biological processes and those contributed by our DNA symbionts.

## 5. Patents

This work is the subject of patent application *Endogenous Retrovirus-K (ERVK) encodes an alternate envelope protein*, WO 2019 075562 A1, 17 October 2018.

## Supplementary Materials

The following are available online at https://www.mdpi.com/2073-4409/9/7/1584/s1, Figure S1: ERVK CTXLP and its similarity with Conus-derived conotoxins, Figure S2: ERVK CTXLP similarity with NPV conotoxin-like proteins, Figure S3: Validation of the custom ERVK CTXLP antibody used in this study, Figure S4: ERVK CTXLP variants in humans, primates and murine model, Figure S5: ERVK CTXLP can localize to different cellular compartments, particularly in cancer cells, Figure S6: ERVK CTXLP is enhanced in the motor cortex of patients with ALS and associated with necroptosis, Figure S7: Identification of transcripts encoding ERVK CTXLP and Env in human disease states, Figure S8: ERVK CTXLP protein expression is associated with demyelinated lesions in spinal cord tissues from patients with ALS, Figure S9: Nogo-A expression in CTXLP+ oligodendrocyte precursors may limit spinal cord remyelination in ALS, Table S1: Proteins and peptides from various organisms and their respective accession numbers used in comparison with the CTXLP cysteine-rich motif, Table S2: ERVK HML-2 insertions in humans and their chromosomal location examined for an intact Rec and/or CTXLP ORF, along with any known disease associations, Table S3: Demographics of patients and tissue sample source, Table S4: Primary and secondary antibodies used, Supplementary methods (Bioinformatics, RNA seq analysis, Orthologues and paralogues analysis, Ethics statement, Diagnosis and demographics of patient samples, Immunohistochemistry of autopsy tissue, Solochrome cyanine staining, Cell culture and treatments, Immunoprecipitation and cell treatments, Immunocytochemistry, Transient transfections, Western Blotting, Quantitative PCR, and Chromatin immunoprecipitation (ChIP)).
