# Supplementary material for: Pro-Inflammatory Signaling Upregulates a Neurotoxic Conotoxin-Like Protein Encrypted Within Human Endogenous Retrovirus-K
Source: Cells. 2020 Jun 30;9(7):1584. doi: 10.3390/cells9071584 (PMC7407490; doi:10.3390/cells9071584)
Supplement: Supplementary file 1 [file cells-09-01584-s001.pdf]

Supplementary information

Table S1: Proteins, peptides and genomes from various organisms and their respective accession numbers used in comparison with CTXLP cysteine-rich motif.

Protein/Peptide	Organism	Uniprot Accession #
Conotoxins	*Conus* species	conus name:conotoxin[a]
Viral conotoxin-like proteins	NPV	nuclear polyhedrosis virus toxin[b]
Guanxitoxin-2	Spider	P84837.1
Guanxitoxin-1D	Spider	P84836.1
Hainantoxin-I	Spider	D2Y1X6.1
Hainantoxin-III	Spider	D2Y1X9.1
Hainantoxin-IV	Spider	1NIY_A
Hainantoxin-V	Spider	P60975.1
Hanatoxin-1	Spider	P56852.1
Hanatoxin-2	Spider	P56853.1
Sgtx	Spider	1LA4_A
Grammotoxin	Spider	P60590.2
Huwentoxin-I	Spider	P56676.2
Huwentoxin-X	Spider	P68424.2
Agouti-related peptide	Human	O00253.1
Agouti-signalling protein	Human	1Y7K_A
VEGF-A	Human	P15692.2
VEGF-B	Human	P49765.2
VEGF-C	Human	CAA63907.1
VEGF-D	Human	BAA24264.1
VEGF-E	Human	ABA00650.1
VEGF-F	Snake	1WQ8_A
Placental Growth Factor	Human	AAH07789.1
Tat	HIV-1	CCD30501.1
Tat	HIV-2	AAA76845.1
Tax	HTLV-1	BAD95659.1
Tax	HTLV-2	AFC76143.1
Tax	HTLV-3	Q0R5R1.1
Envelope	HTLV-4	CAA29690.1
Envelope	Jaagsiekte Sheep Retrovirus	AAK38688.1
**Genome**		**NCBI Accession #**
HIV-1		NC_001802.1
HIV-2		NC_001722.1
HTLV-1		NC_001436.1
HTLV-2		NC_001488
HTLV-3		EU649782
HTLV-4		X06391
MMTV		NC_001503
ERVW		NM_001130925.2
ERVW		NM_014590.3
**Genome**		**Repbase Accession #**
ERVH		HERVH
ERVK (HLM-2)		HERVK
ERVW		HERV17
**Genome**		**HIV Sequence Database**

Supplementary information

170 representative HIV strains	https://www.hiv.lanl.gov/content/sequence/NEWALIGN/align.html

[a] Uniprot: https://www.uniprot.org/uniprot/?query=conus+name%3Aconotoxin&sort=score

[b] Uniprot: https://www.uniprot.org/uniprot/?query=nuclear+polyhedrosis+virus+toxin&sort=score

Table S2: ERVK HML-2 insertions in humans and their chromosomal location examined for an intact Rec and/or CTXLP ORF, along with any known disease associations.

Accession number	Genomic location	ERVK insertion	Intact Rec?	Intact Conotoxin?
JN675007	1p31.1	**ERVK-1[a]_HML-2_1p31.1_75842771**	No	**Yes**
JN675010	1p36.21b	ERVK-76[b]_HML-2_1p36.21b_ 13458305	No	No
JN675011	1p36.21c	ERVK-76[b]_HML-2_1p36.21c_13678850	No	No
JN675013	1q23.3	**ERVK-18[a]_HML-2_1q23.3_ 160660575**	No	**Yes**
JN675014	1q22	**ERVK-7[a]_HML-2_1q22_ 155596457**	No	**Yes**
JN675015	1q24.1	ERVK-12[b]_HML-2_1q24.1_ 166574603	No	No
JN675016	1q32.2	ERVK_ HML-2_1q32.2_ 207808457	No	No
JN675018	2q21.1	**ERVK_HML-2_2q21.1_ 130719538**	No	**Yes**
JN675019	3p12.3	ERVK_HML-2_3p12.3_ 75600465	No	No
JN675020	3p25.3	ERVK-2[a,b]_HML-2_3p25.3_9889346	No	No
JN675021	3q12.3	**ERVK-5[a]_HML-2_3q12.3_ 101410737**	No	**Yes**
JN675022	3q13.2	**ERVK-3[a]_HML-2_3q13.2_112743479**	No	**Yes**
JN675023	3q21.2	**ERVK-4[a]_HML-2_3q21.2_ 125609302**	No	**Yes**
JN675025	3q27.2	**ERVK-11[a]_HML-2_3q27.2_185280336**	No	**Yes**
JN675026	4p16.1a	ERVK-17[b]_HML-2_4p16.1a_9123515	No	No
JN675027	4p16.1b	ERVK-50c[b]_HML-2_4p16.1b_9659588	No	No
JN675029	4p16.3b	ERVK-7[b]_HML-2_4p16.3b_3980069	No	No
JN675030	4q13.2	ERVK_HML-2_4q13.2_463709	No	No
JN675032	4q32.3	ERVK-13[a]_HML-2_4q32.3_5916840	No	No
JN675034	5p12	ERVK_HML-2_5p12_46000159	No	No
JN675035	5p13.3	**ERVK-104[b]_HML-2_5p13.3_30487114**	No	**Yes**
JN675036	5q33.2	ERVK-18b[b]_HML-2_5q33.2_154016502	No	No
JN675037	5q33.3	**ERVK-10 [a]_HML-2_5q33.3_156084717**	No	**Yes**
JN675039	6p21.1	ERVK-OLD35587b_HML-2_6p22.1_42861409	No	No
JN675040	6p22.1	ERVK-69_HML-2_6p22.128650367	No	No
JN675041	6q14.1	**ERVK-9[a]_HML-2_6q14.1_78427019**	**Yes**	**Yes**
JN675043	7p22.1a	**ERVK-14[a]_HML-2_4622057**	**Yes**	**Yes**
JN675044	7p22.1b	**ERVK-14[a]_HML-2_4630561**	**Yes**	**Yes**
JN675049	8p23.1a	**ERVK-8[a]_HML-2_8p23.1a _7355397**	No	**Yes**
JN675050	8p23.1b	ERVK-27[b]_HML-2_8p23.1b_8054700	No	No
JN675051	8p23.1c	ERVK_HML-2_8p23.1_12073970	No	No
JN675052	8p23.1d	ERVKOLD130352[b]_HML-2_8p23.1d_12316492 1	No	No
JN675053	8q11.1	ERVK-70[b]_HML-2_8q11.1_47175650	No	No
JN675057	9q34.11	ERVK-31[b]_HML-2_9q34.11_131612515	No	No
JN675058	10p12.1	**ERVK-103[b]_HML-2_10p12.1_27182399**	No	**Yes**
JN675059	10p14	ERVK-16[a]_HML-2_10p14_6867109	No	No

Supplementary information

JN675060	10q24.2	ERVK-17[a]_HML-2_10q24.2_101580569	No	No
JN675061	11p15.4	ERVK3-4[a]_HML-2_11p15.4_ 3468656	No	No
JN675062	11q12.1	ERVK_HML-2_ 11q12.1_58767448	No	No
JN675063	11q12.3	ERVK-OLDAC004127[b]_HML-2_11q12.3_ 62135963	No	No
JN675064	11q22.1	**ERVK-25[a]_HML-2_11q22.1_ 101565794**	**Yes**	**Yes**
JN675065	11q23.3	**ERVK-20[a]_HML-2_ 11q23.3_118591724**	No	**Yes**
JN675066	12p11.1	ERVK-50E[b]_HML-2_12p11.1_ 34772555	No	No
JN675067	12q13.2	**ERVK_HML-2_12q13.2_ 55727215**	No	**Yes**
JN675068	12q14.1	**ERVK-21[b]_HML-2_12q14.1_ 58721242**	No	**Yes**
JN675073	15q25.2	ERVK_HML-2_15q25.2_84829020	No	No
JN675074	16p11.2	**ERVK_HML-2_16p11.2_ 34231474**	No	**Yes**
JN675075	17p13.1	ERVK_HML-2_17p13.1_7960357	No	No
JN675076	19p12a	ERVK52[b]_HML-2_ 19p12a _20387400	No	No
JN675077	19p12b	**ERVK113[b]_HML-2_19p12b_21841536**	**Yes**	**Yes**
JN675078	19p12c	**ERVK51[b]_HML-2_19p12c_22757824**	No	**Yes**
JN675080	19q11	ERVK-19[b]_HML-2_19q11_228128498	Yes	No
JN675081	19q13.12a	ERVK_HML-2_19q13.12a_36063207	No	No
JN675082	19q13.12b	ERVKOLD12309_HML-2_19q13.12b_37597549	No	No
JN675083	19q13.41	ERVK3-6[a]_HML-2_19q13.41_53248274	No	No
JN675084	19q13.42	LTR13[b]_HML-2__19q13.42_53862348	No	No
JN675085	20q11.22	**ERVK59[b]_HML-2_20q11.22_32714750**	No	**Yes**
JN675086	21q21.1	**ERVK-23[a]_HML-2_21q21.1_19933916**	No	**Yes**
JN675087	22q11.21	**ERVK-24[a]_HML-2_22q11.21_18926187**	No	**Yes**
JN675088	22q11.23	ERVK-KOLD345b_HML-2_22q11.23_23879930	No	No
JN675090	Xq11.1	ERVK_HML-2_Xq11.1_61959549	No	No
JN675094	Yp11.2	ERVK_HML-2_ Yp11.2_6 826441	No	No

Disease associations: MS (yellow,) MS (No CTXLP; pale yellow), Cancer (Green), Cancer (No CTXLP; dark green), Schizophrenia (blue).

a) Mayer, J., Blomberg, J., & Seal, R. L. (2011). A revised nomenclature for transcribed human endogenous retroviral loci. Mobile DNA, 2(1), 7.

b) Subramanian, R. P., Wildschutte, J. H., Russo, C., & Coffin, J. M. (2011). Identification, characterization, and comparative genomic distribution of the HERV-K (HML-2) group of human endogenous retroviruses. Retrovirology, 8(1), 90.

Supplementary information

Table S3: Demographics of patients and tissue sample source.

Case	Diagnosis	Cause of death	Tissue	Age	Gender	PMI (hours)	Tissue bank	WB	Confocal
2712	ALS	ALS	BA6, CC	67	F	12.5	NBB	●	
2776	ALS	ALS (PBP)	BA6, CC	76	F	8.6	NBB	●	
4501	ALS	ALS	BA4, BA6, CC	58	F	15.1	NBB	●	●
4739	ALS	ALS	BA6, CC	61	F	7.5	NBB	●	●
4755	ALS	ALS	BA6, CC, LC	40	M	15.6	NBB	●	●
4766	ALS	ALS	BA4, BA6, CC, LC	82	F	12.1	NBB	●	●
5187	ALS	ALS, HT, CUTI	BA6, CC	69	M	13.2	NBB	●	
5212	ALS	ALS	BA4, BA6, CC	50	M	21	NBB	●	●
5215	ALS	ALS	BA4, BA6, CC	59	M	12.5	NBB	●	●
5216	ALS	ALS	BA4, BA6, CC	53	M	16.8	NBB	●	●
100034	ALS	ALS (HR, RF)	BA4, BA6, CC	62	M	1.66	VABT	●	●
100039	ALS	ALS (RF)	CC	50	M	5	VABT	●	
110007	ALS	ALS (RF)	CC	80	M	1.25	VABT		
110009	ALS	ALS	BA4, BA6, CC	84	M	3.58	VABT	●	●
110011	ALS	ALS	BA4, BA6, CC, LC	83	M	2.5	VABT	●	●
110036	ALS	ALS	BA4, BA6	85	M	2.5	VABT	●	

Supplementary information

120011	ALS	ALS	BA4, BA6, CC	58	F	1	VABT	●	
120018	ALS	ALS	BA4, BA6, CC, LC	83	M	0.25	VABT	●	●
120021	ALS	ALS (RF)	BA4, BA6	70	M	0.5	VABT	●	
3221	Normal	COPD	BA4, BA6	90	M	17.8	NBB	●	●
3298	Normal	Hypoxia	BA6	79	M	20	NBB	●	
3359	Normal	Heart attack, stroke	BA4, BA6	86	M	14.5	NBB		●
3371	Normal	LC	BA6	52	M	16	NBB	●	●
3504	Normal	Normal aging	BA6	80	M	11	NBB	●	
3565	Normal	Cardiomyopathy	BA4, BA6	76	M	11	NBB	●	●
4294	Normal	PC, HT	BA6	80	M	19.2	NBB	●	●
4307	Normal	SC, Rn F, COPD, DB, HT	BA4, BA6	84	M	11.8	NBB		●
4514	Normal	LC, COPD	BA6	66	M	17.3	NBB	●	
4660	Normal	PC, DB, HT	BA4, BA6	73	F	18.5	NBB	●	●
5452	Normal	Normal aging	CC, LC	67	M	23	NBB	●	●
5572	Normal	Normal aging	CC	70	F	24	NBB	●	
5611	Normal	Normal aging	CC	50	F	15	NBB	●	
5617	Normal	Normal aging	CC, LC	59	M	10	NBB	●	●
5628	Normal	Normal aging	CC	54	F	27	NBB	●	
5656	Normal	Normal aging	CC	51	F	21	NBB	●	●
5762	Normal	Normal aging	CC	39	F	19	NBB	●	
100012	Normal	DADL	CC, LC	81	F	4	VABT	●	●
110005	Normal	PE	BA4, BA6, CC, LC	67	M	2.75	VABT	●	●
110023	Normal	CS	CC, LC	71	M	2.42	VABT	●	●

Brain bank
NBB: Neurobiobank

Supplementary information

VABT: Veterans affairs biorepository tissues

Disease
ALS: Amyotrophic lateral sclerosis
COPD: Chronic obstructive pulmonary disease
CS: Cardiogenic shock
CUTI: Chronic urinary tract infection
DADL: Diffuse alveolar damage of the lungs
DB: Diabetes
HT: Hypertension
HF: Heart failure
LC: Lung cancer
PBP: Progressive bulbar palsy
PC: Pancreatic cancer
PE: Pulmonary edema
RF: Respiratory failure
Rn F: Renal failure
SC: Stomach cancer

Tissue
BA4: Brodmann area 4 motor cortex
BA6: Brodmann area 6 prefrontal cortex
CC: Cervical spinal cord
LC: Lumbar spinal cord

Other
WB: Western blot
PMI: post-mortem interval

Supplementary information

Table S4: Primary and secondary antibodies used.

Primary antibody	Source	Product number	Host
β-actin	ThermoFisher Scienctific, Rockford, IL, USA	MA5-15739	Mouse monoclonal
β-actin	Abcam, Cambridge, MA, USA	ab13822	Chicken polyclonal
Active and pro-Caspase-3 (31A1067)	Abcam, Cambridge, MA, USA	ab1355	Mouse monoclonal
CX3CL1	Abcam, Cambridge, MA, USA	ab89229	Mouse monoclonal
Endogenous Retrovirus-K, Human Conotoxin-like Protein (serum)	Custom	Custom	Rabbit polyclonal
Endogenous Retrovirus-K, Human Conotoxin-like Protein (affinity purified)	Custom	Custom	Rabbit polyclonal
Endogenous Retrovirus-K, Human Envelope Protein	LifeSpan BioSciences, Inc.	LS-C65286	Mouse monoclonal
Endogenous Retrovirus-K, Human Reverse Transcriptase	Abnova	H00002087-A01	Mouse polyclonal
MAP2	Novus Biologicals, Littleton, CO	NB300-213	Chicken polyclonal
MLKL (3B2)	Santa Cruz Biotechnology, Dallas, TX	sc-293201	Mouse monoclonal
Myelin associated glycoprotein (clone 513)	Millipore, Temecula, CA	MAB1567	Mouse monoclonal
Myelin basic protein	Millipore, Temecula, CA	AB9348	Chicken polyclonal
N-type Ca++ CP α1B (A-2) [Cav2.2]	Santa Cruz Biotechnology, Dallas, TX	sc-377489	Mouse monoclonal
NF-κB p65	Abcam, Cambridge, MA	ab243	Sheep polyclonal
Nogo-A (aa566-748)	Novus Biologicals, Oakville, ON	AF3515	Sheep polyclonal
Olig-1	Millipore, Temecula, CA	MAB5540	Mouse monoclonal
TCF-4 (clone 6H5-3)	Millipore, Temecula, CA	05-511	Mouse monoclonal
βIII tubulin	Abcam, Cambridge, MA	ab41489	Chicken polyclonal

Supplementary information

Secondary antibody	Source	Product number	Host
ALEXA FLUOR 488 Goat anti-mouse IgG	Life Technologies, Inc., Burlington, ON	A11017	Goat
ALEXA FLUOR 594 Goat anti-mouse IgG	Life Technologies, Inc., Burlington, ON	A11020	Goat
ALEXA FLUOR 647 Goat anti-mouse IgG	Life Technologies, Inc., Burlington, ON	A21237	Goat
ALEXA FLUOR 488 Goat anti-rabbit IgG	Life Technologies, Inc., Burlington, ON	A11070	Goat
ALEXA FLUOR 594 Goat anti-rabbit IgG	Life Technologies, Inc., Burlington, ON	A11072	Goat
ALEXA FLUOR 647 Goat anti-rabbit IgG	Life Technologies, Inc., Burlington, ON	A21246	Goat
ALEXA FLUOR 594 Donkey anti-sheep IgG	Life Technologies, Inc., Burlington, ON	A11016	Donkey
ALEXA FLUOR 488 Goat anti-chicken IgG	Life Technologies, Inc., Burlington, ON	A11039	Goat
ALEXA FLUOR 647 Goat anti-chicken IgG	Life Technologies, Inc., Burlington, ON	A21449	Goat
ALEXA FLUOR 647 Donkey anti-rat IgG	Life Technologies, Inc., Burlington, ON	A11007	Donkey
Goat anti-rabbit (H+L) HRP Conjugate	Biorad	#170-6515	Goat
Goat anti-mouse (H+L) HRP Conjugate	Biorad	#170-6516	Goat

Supplementary information

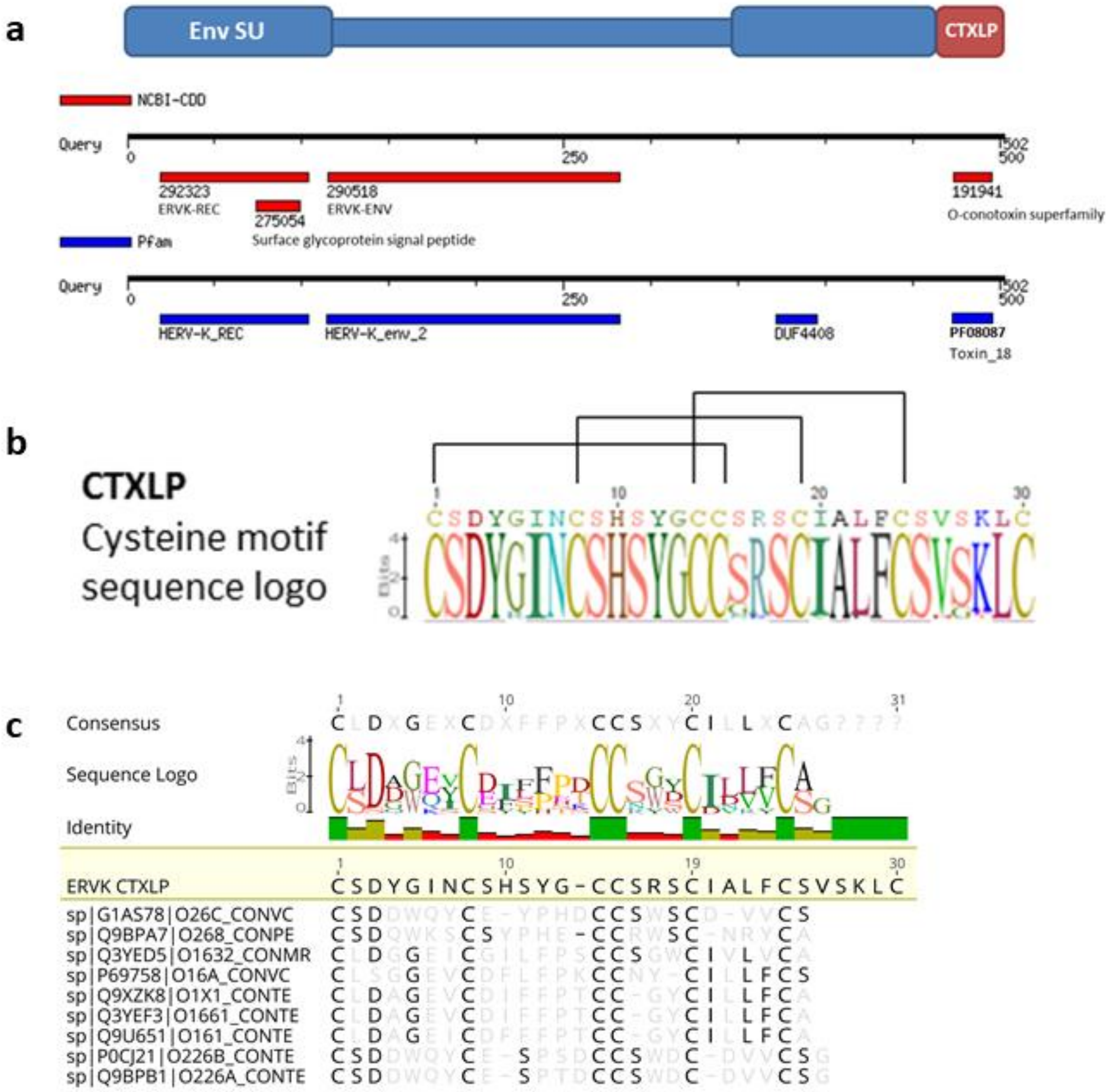

Supplementary information

Figure S1: ERVK CTXLP and its similarity with *Conus*-derived conotoxins. (**A**) Schematic representation of CTXLP and amino acid sequence similarities found using NCBI-CDD and Pfam databases. The SU subunit of CTXLP is blue and the omega conotoxin domain is in red. The wider portions of the diagram represent the ordered regions of the proteins and the narrow region represents the disordered region as predicted by Eukaryotic Linear Motif (ELM) resource [1]. (**B**) Predicted inhibitor cysteine knot fold of ERVK CTXLP cysteine-rich peptide. Disulfide bonds connect cysteine 1 to cysteine 4, cysteine 2 to cysteine 5, and cysteine 3 to cysteine 6, resulting in an inhibitor cysteine knot fold. (**C**) Alignment of ERVK CTXLP and representative O-conotoxin sequences with identity scores above 40%, from cone snail species *Conus victoriae, Conus pennaceus, Conus marmoreus* and *Conus textile*. Note the conservation of the characteristic C-C-CC-C-C knottin folding motif [2].

Supplementary information

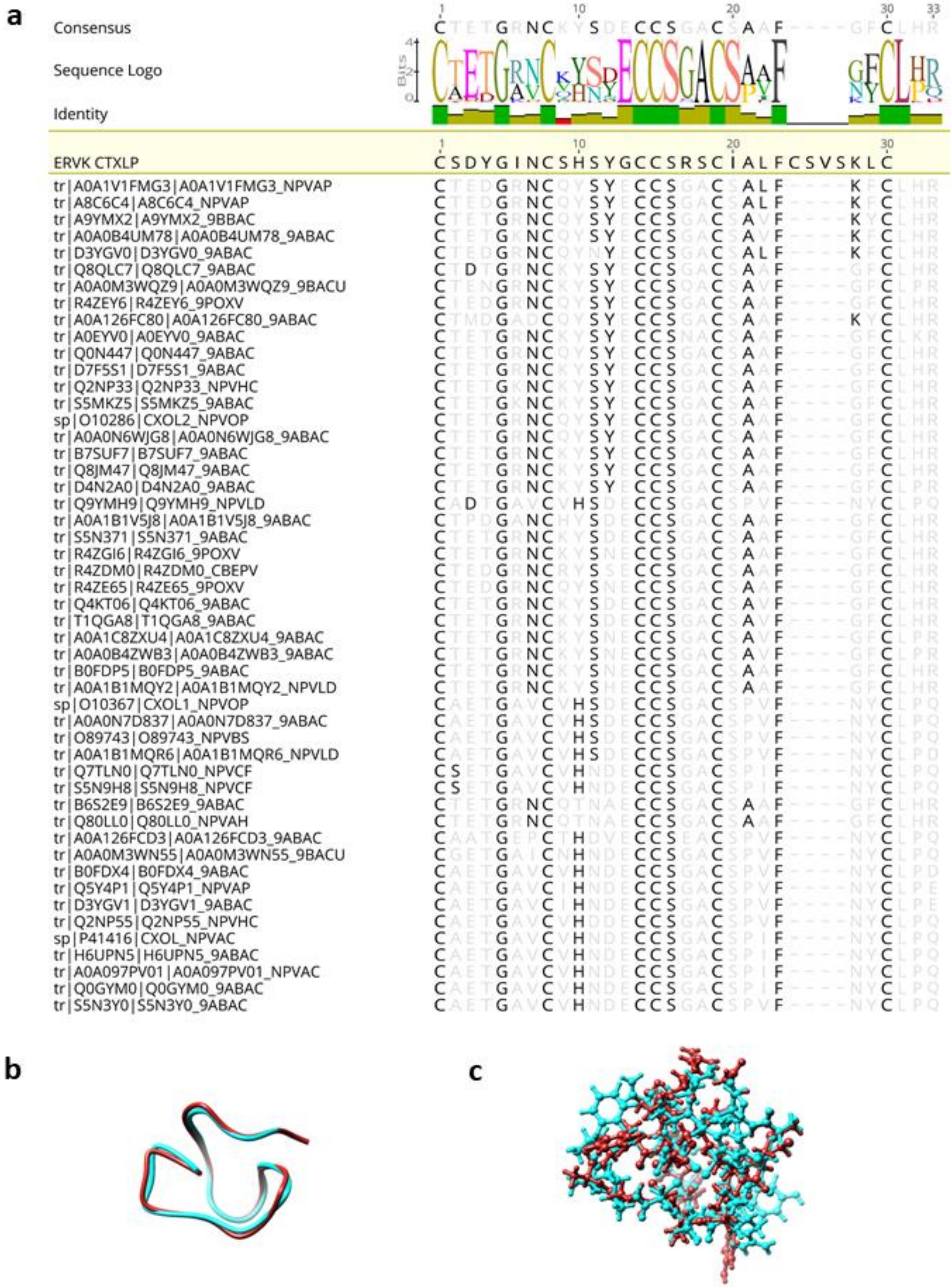

Supplementary information

Figure S2: ERVK CTXLP similarity with NPV conotoxin-like proteins. (**A**) Alignment and sequence logo of endogenous retrovirus K-113 conotoxin-like protein (ERVK-113 CTXLP) cysteine-rich motif and Nuclear Polyhedrosis Virus (NPV) conotoxin-like protein sequences. Sequences were aligned, and sequence logo was assessed using Geneious Prime software. Note the conserved C-G-NC-SY-CCS-C-ALF-C sequence logo in these viral conotoxin-like proteins. (**B** and **C**) Aligned overlap of the predicted backbone (**B**) and peptide (**C**) structures of viral conotoxin-like protein backbones from ERVK-113 and *Ecotropis obliqua* NPV. Knotter1D3D was used to predict the structures of putative ERVK-113 CTXLP domain (blue) and *Ecotropis obliqua* NPV CTXLP domain (red). Structure alignment is based on sequence alignment and was prepared using UCSF Chimera software [3].

Supplementary information

a

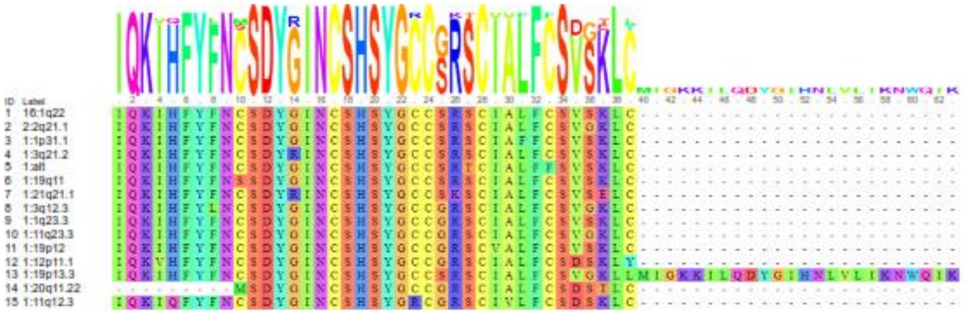

b

Orthologous ERVK fragments identified by BLAST				
	H. sapiens	*P. troglodytes*	*G. gorilla*	*C. atys*
H. sapiens	**7358**	6060	5853	2585
P. troglodytes	6060	**7389**	5812	2581
G. gorilla	5853	5812	**6504**	2562
C. atys	2585	2581	2565	**7411**

Number of PF08087* and PF13804* Loci by Species		
Species	CTXLP	Env
H. sapiens	28	383
P. troglodytes	33	402
G. gorilla	39	318
C. atys	31	379

c

```
Representative Sequences from Each Species
gorilla     526948   IQKIHFYFNCSDYGINCSHSYGCCGRSCIALFCSVGKLC
mangabey    1353842  IQKIHFYFNCSDYGINCSHSYSLCGRSCIALFCSDSKLC
chimpanzee  13529    IQKIHFYFNCSDYGINCSHSYGCCGRSCIALFCSVSKLC
human       1227     IQKIHFYFNCSDYGINCSHSYGCCSRSCIALFCSVGKLC
```

d

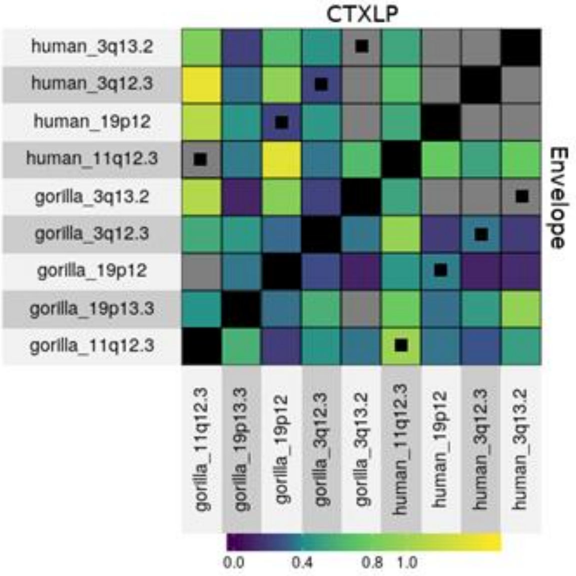

e

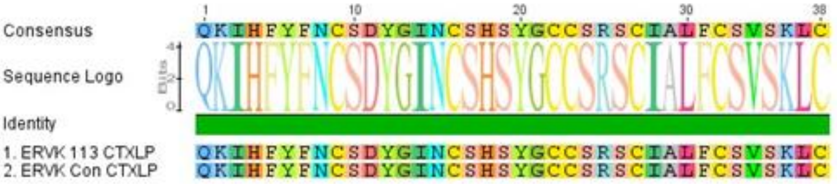

Supplementary information

Figure S3: ERVK CTXLP variants in humans, primates and murine model. (**A**) CTXLP variants in the humans, based on genome build GRCh38. In the alignment above, we see that the first variant is the most common, with 16 copies (representing ERVK-3, -7, -8, -9, -10, -11, -14, -14(b) -21, -24, -25, -103, -104 and -113), followed by the second with 2 (ERVK-5 & -20). All the following ones are present as unique copies. The last three sequences with a deletion or a mutated cysteine residue are likely to be non-functional. (**B**) ERVK proviruses are not human specific, and several ERVK insertions are shared with other primates [4], as indicated by the number of ERVK, CTXLP (PF08087) and Env (PF13804) positive loci in primates. (**C**) Examination of CTXLP encoding loci in three non-human primate genomes, *Pan troglodytes* (Common chimpanzee), *Gorilla gorilla gorilla* (Western lowland gorilla), and *Cercocebus atys* (Sooty Mangabey), as well as humans reveals orthologous loci and conservation of the Toxin_18 cysteine motif (yellow). The name of each sequence is the retroelement identification number of the locus encoding it. Representative sequences from each from each primate species highlights the degree of conservation in the Toxin_18 cysteine motif. Some CTXLP-encoding loci, such as ERVK-18 and ERVK-20, have orthologues in non-human primates, suggesting that CTXLP is an evolutionarily conserved protein of ERVK. (**D**) Different mutational patterns between orthologues and paralogues of ERVK env genes. Represented is a combined set of heatmaps generated by superheat from frames 0 (CTXLP) and 1 (Envelope) of the human and gorilla orthologues ORFs, which where both are positive for Toxin_18 positive (CTXLP). The sequences which are orthologues are indicated by black squares in the center of each space (paralogues do not have black squares). Blue is an ω (dN/dS ratio) less than 1, indicating purifying selection and similarity between the sequences. Yellow is an ω more than 1, indicating diversifying selection and dissimilarity between the sequences. Grey indicates that ω could not be computed (in all cases dS = 0, indicating no synonymous differences between the two sequences). Sequences which are identical along the diagonal are blacked out. The cytological bands are based on the human genome, with gorilla designations indicating their respective human orthologue/paralogue coordinate. There exists both orthologous (sequences shared by a common ancestor) and paralogous (sequences derived from an ancestral gene/virus) ERVK insertions in humans and gorilla. When comparing the evolutionary pressures on the ERVK SU (reference reading frame) versus CTXLP (-1 reading frame)

sequences, there is more conservation of the CTXLP domain between human-gorilla orthologues than for paralogues in humans, as compared with the SU reading frame. Despite this evidence for diversifying selection of CTXLP sequences in humans, CTXLP domains from all primates maintain a strong degree of conservation of the core cysteine-rich motif (panel c). (**E**) Alignment of ERVK113 CTXLP sequence and the ERVK Consensus sequence found in the ERVK envelope transgenic mice[5]. Note the similarity and retention of key cysteine motif.

Supplementary information

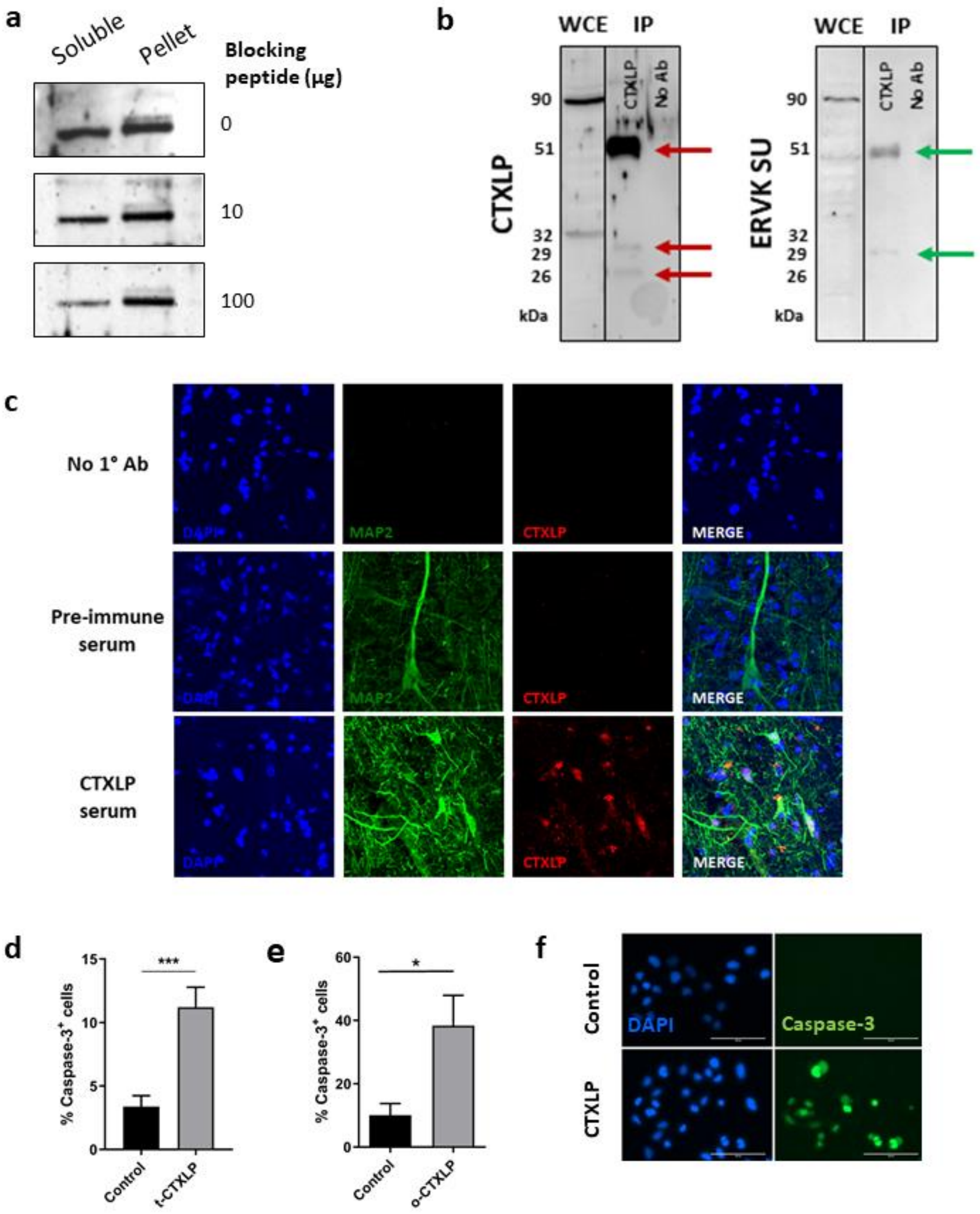

Supplementary information

Figure S4: Validation of the custom ERVK CTXLP antibody used in this study. (**A**) Three identical soluble (lysis buffer extracted) and insoluble (RIPA-extracted) SVGA lysate western blots were incubated with custom rabbit ERVK CTXLP antibody in the presence of purified CTXLP blocking peptide at 0, 10 and 100 μg/mL This peptide was the immunogen used in the generation of the CTXLP-specific polyclonal sera. High dose CTXLP peptide competitively blocked the custom antibody detection of the major 90 kDa form of CTXLP in the cell lysates. (**B**) Matched NCCIT WCE and IP fractions (as described in A) were blotted for CTXLP and Env surface unit (SU) expression. Note that similar bands contain both CTXLP (red arrows) and SU (green arrows) epitopes, suggesting the generation of unmodified SU-CTXLP fusion protein isoforms at 29 and 51 kDa, and potentially larger post-translationally modified forms between 90 -110 kDa. (**C**) Human autopsied tissue from a patient with ALS was stained using a commercial MAP-2 antibody in conjunction with either rabbit pre-immune serum or anti-CTXLP serum collected 90 days post immunization against the ERVK CTXLP core cysteine-rich peptide. The rabbit immunization protocol for generation of a polyclonal antibody against the ERVK CTXLP domain yields specific and consistent tissue staining patterning, unlike the pre-immune sera. A no primary antibody control depicts the background level of fluorescence in each channel. DAPI staining indicates the presence of cell nuclei within tissues. (**D**–**F**) CTXLP exposure enhances cleaved caspase-3 levels in SVGA cells, indicative of cellular apoptosis. SVGA cells were transfected with empty vector and lipofectamine LTX (control) or a pcDNA3.1 CTXLP-expressing vector (t-CTXLP). At 24 h, live cell images were taken using EVOS microscope (panel D–F). CTXLP-transfection markedly enhanced cellular cleaved capase-3 levels after 24 hours (panel D, $n = 3$, $*p < 0.001$). Alternatively, cells were treated with CTXLP proteins isolated from ERVK-producing NCCIT cells via immunoprecipitation (E–F). Our overlay model simulates conditions wherein CTXLP would enter the cell from the outside and possibly exert its effects by binding to cell surface receptors (such as voltage-gated calcium channels (VGCCs), as do conotoxins [6]). SVGA cells were cultured with 5 μl of immunoprecipitation (IP) buffer or CTXLP-IP fraction (overlaid, o-CTXLP) (panel E, 250–500 cells per trial, $n = 3$, $*p < 0.05$). Cells treated with extracted CTXLP also had increased caspase-3 cleavage as compared to control cells, demonstrating that CTXLP was toxic to astrocytes even at vanishingly low protein concentrations. The percentage of

positive cells was determined by quantification of the number of cells displaying cleaved caspase-3 over total cells in micrographs. Representative images are shown (panel F). Together, this suggests that exposure to extracellular CTXLP and/or cellular production of CTXLP in vivo may be toxic to cells.

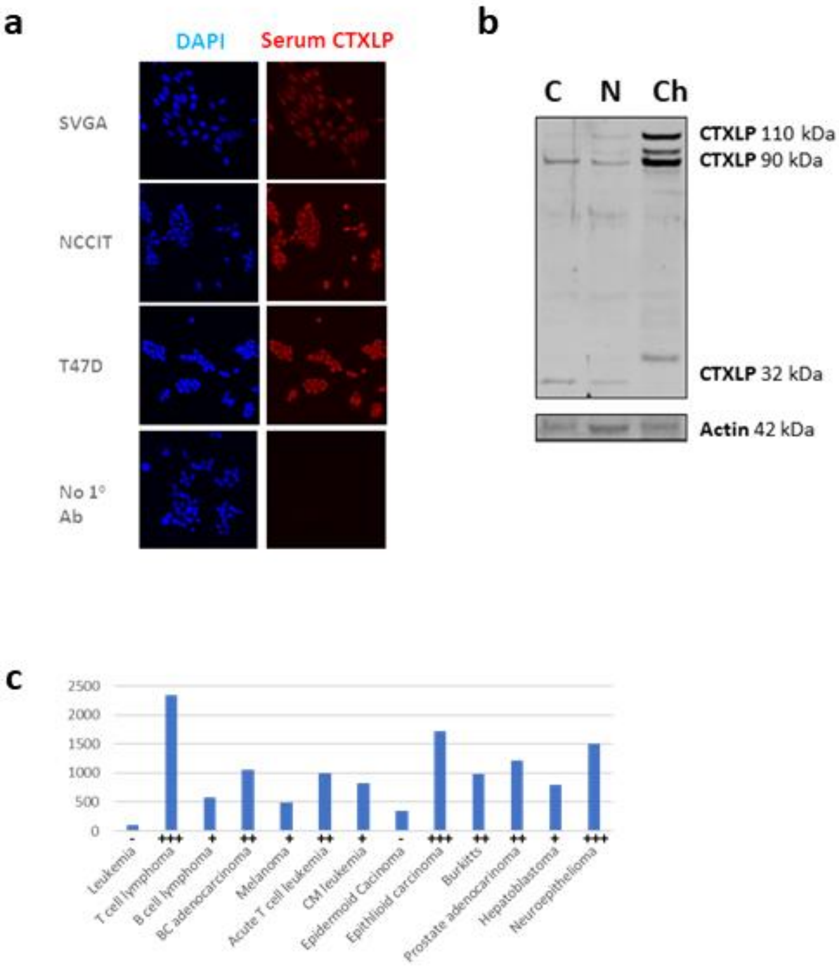

Supplementary information

Figure S5: ERVK CTXLP can localize to different cellular compartments, particularly in cancer cells. (**A**) Cancer cells express greater levels of CTXLP as compared to non-cancer cells. Prototypic cell lines for teratocarcinoma (NCCIT) and breast cancer (T47D) were examined for CTXLP expression as compared to astrocytic SVGA cells using confocal microscopy. No antibody negative control is to show that specificity of CTXLP (red) staining requires an antibody targeting ERVK CTXLP. Nuclei are shown in blue using a DAPI stain. (**B**) Human cells lines vary in their expression and cellular localization of CTXLP protein, based on cancer status. ERVK-producing NCCIT cells, in the absence of stimuli, were subject to cytoplasmic (C), nuclear (N) and chromatin (Chr) cell fractionation. NCCIT cells exhibited CTXLP protein expression in all cell lysate fractions, but a clear enrichment of large isoforms of CTXLP (90-110 kDa) in the chromatin fraction (n = 4). (**C**) CTXLP expression in G-Bioscience Ready-to-screen cancer cell line blot (TB55) was screened for CTXLP expression (blue bars) normalized to β-actin loading control. Enhanced CTXLP expression in noted is several cancer types, including T cell lymphoma, epithelioid carcinoma, neuroepithelioma and prostate cancer.

Supplementary information

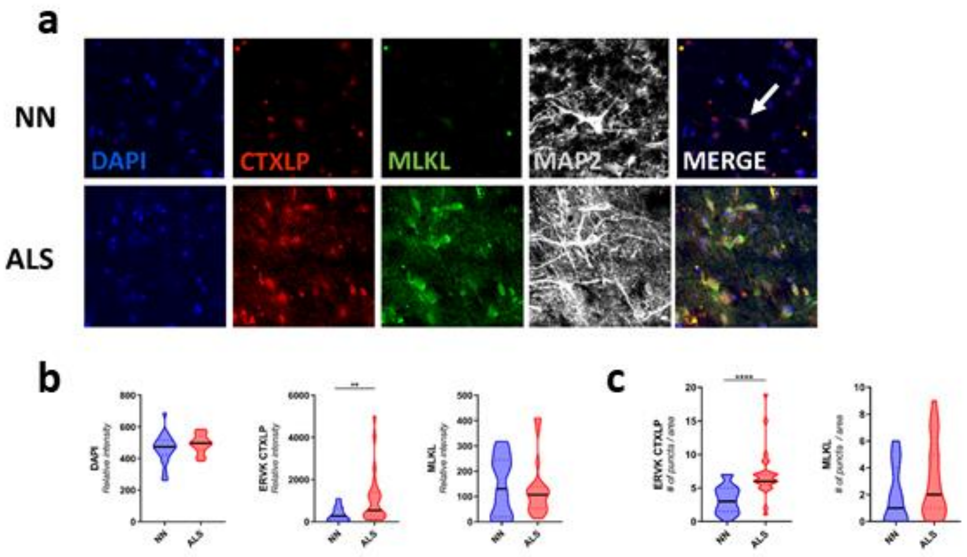

Supplementary information

Figure S6: ERVK CTXLP is enhanced in the motor cortex of patients with ALS and associated with necroptosis. (**A**) ERVK CTXLP levels are enhanced in motor cortex brain tissues of patients with ALS, as measured by confocal microscopy. Representative 40x confocal micrographs of ERVK CTXLP (red), MLKL (necroptosis marker, green) and neuronal MAP2 expression (grey) in Brodmann area 4 (BA4, panel A) primary motor cortex tissue of a NN control ($n = 5$) and patient with ALS ($n = 5$). DAPI stain depicts nuclei. Arrow indicates predominantly nuclear CTXLP staining in NN control, in contrast to both nuclear and cytoplasmic CTXLP staining in ALS neurons. (**B**) Violin plots of staining quantification of DAPI, CTXLP and MLKL in NN and ALS cohorts for BA4 tissue. (**C**) Violin plots of protein puncta quantification of CTXLP and MLKL in NN and ALS cohorts for BA4 tissue. Statistical test with unpaired two-tailed t-tests, (** $p < 0.01$, **** $p < 0.0001$, black bars are medians).

Supplementary information

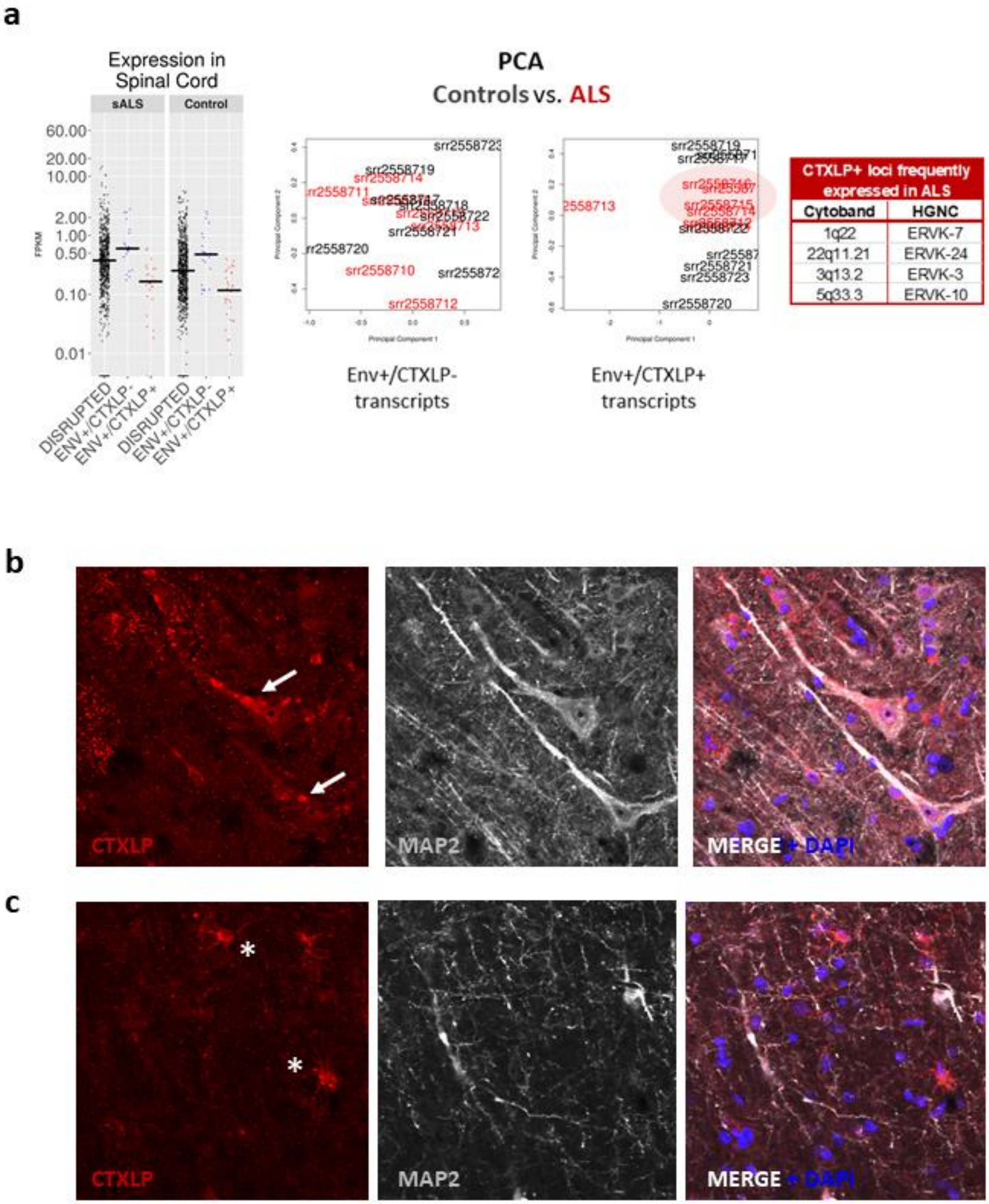

Figure S7: Identification of transcripts encoding ERVK CTXLP and Env in human disease states. (**A**) Per-Locus Differential ERVK Expression. RNA-Seq datasets in the Sequence Read Archive (SRA) used for the analysis of ERVK expression for Amyotrophic Lateral Sclerosis (ALS) was SRP064478. ERVK CTXLP encoding transcripts and CTXLP protein are present in ALS. Re-analysis of RNAseq data[7] in control (right) and sporadic ALS (left) spinal cords for expression of disrupted non-coding (black), Env+/CTXLP- (blue) and Env+/CTXLP+ (red) *env* transcripts. ERVK loci with very low expression are excluded; only loci with a median expression greater than 0 and a mean expression greater than 0.1 are plotted. A lack of differential total ERVK *env* RNA expression in controls versus the ALS cohort was observed for both canonical *env* transcripts and CTXLP-encoding *env* transcripts. Principle component analysis (PCA) reveals ALS patient clustering in terms of $CTXLP^{+}$ transcript expression, with most frequently expressed CTXLP encoding loci indicated. Thus, specific CTXLP loci may drive the expression of CTXLP protein in ALS. (**B**) Representative illustration of CTXLP proteinopathy (white arrow) in ALS motor cortex layer V (panel B). $CTXLP^{+}$ pyramidal neurons exhibit enhanced MAP2 staining in the axonal hillock, indicative of degeneration. In panel C, evidence of the presence of $CTXLP^{+}MAP2^{-}$ cells with astrocytic morphology in ALS are indicated by stars.

Supplementary information

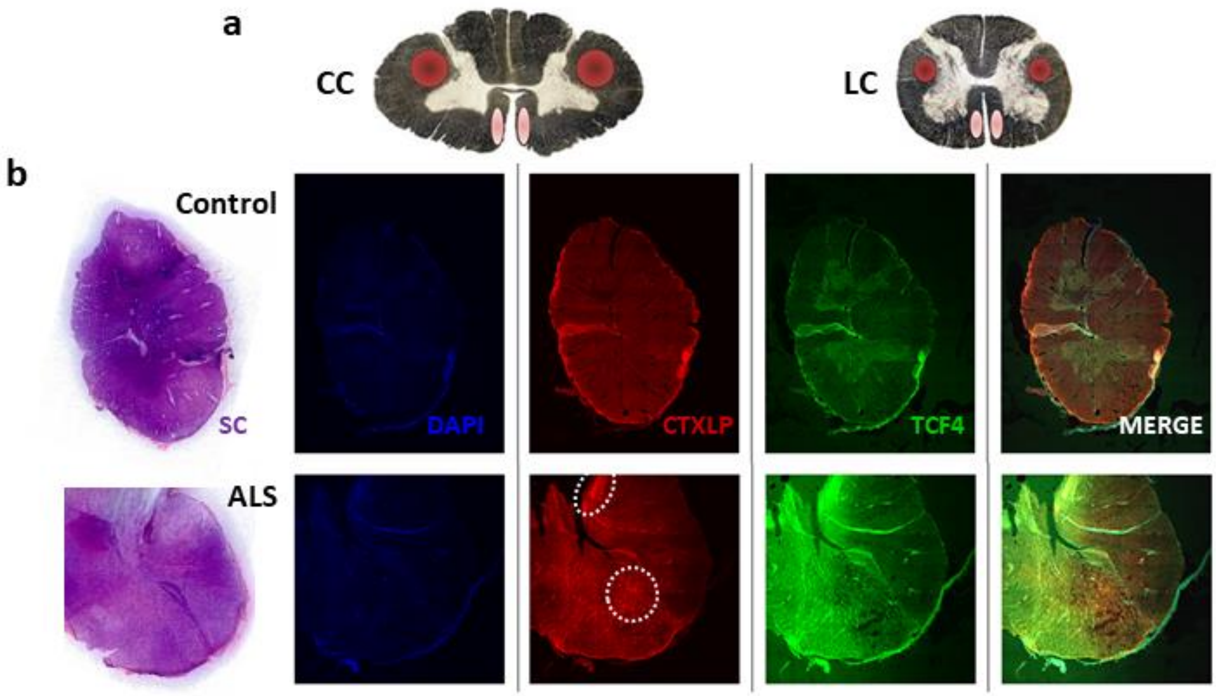

Supplementary information

Figure S8: ERVK CTXLP protein expression is associated with demyelinated lesions in spinal cord tissues from patients with ALS. (**A**) Depiction of motor tracts in cervical spinal cord and lumbar spinal cord (adapted from ibiblio.org). (**B**) ERVK CTXLP levels are enhanced in autopsy cervical spinal cord tissues from patients with ALS, as measured by light and confocal microscopy. Representative 10x confocal micrographs of ERVK CTXLP expression in ex vivo cervical (CC) spinal cord of a neuro-normal control (NN, $n = 5$) and patients with ALS ($n = 5$). Solochrome cyanine (SC) stain (purple) with eosin counterstain (pink) depicts tissue myelination; pale lesions appear in ALS tissues. These lesioned areas exhibited increased CTXLP protein expression (red). Oligodendrocyte precursor marker TCF4 is in green. DAPI stain depicts cellular nuclei. Note: CTXLP expression occurs in either lateral and/or anterior cortical spinal tracts (indicated by white circles).

Supplementary information

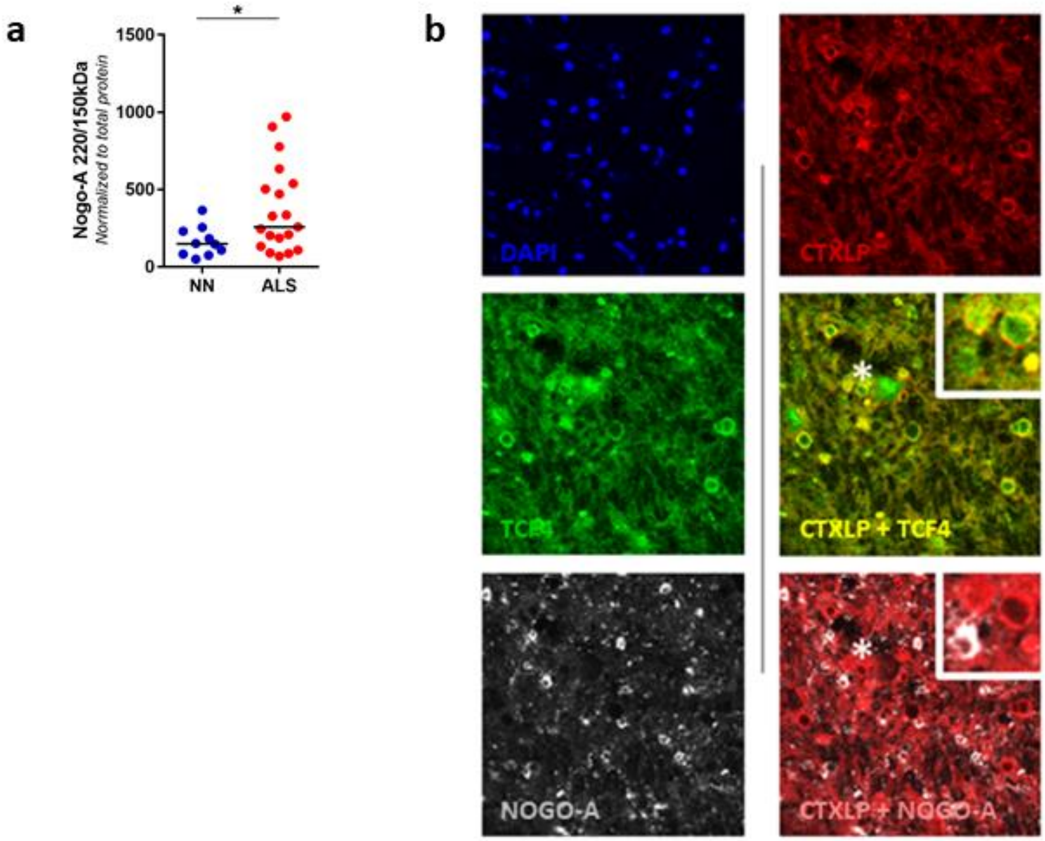

Supplementary information

Figure S9: Nogo-A expression in CTXLP+ oligodendrocyte precursors may limit spinal cord remyelination in ALS. (**A**) Nogo-A levels are enhanced in autopsy spinal cord tissues of patients with ALS, as observed upon western blot analysis quantification of neuro-normal (NN) controls (n = 10) and ALS (n = 19) cervical spinal cord specimens. Statistical test with unpaired two-tailed t-tests, (* $p < 0.05$, black bars are medians). (**B**) ERVK CTXLP+ oligodendrocyte precursors either express myelin inhibitory protein Nogo-A (**Figure 7**), or lie in close proximity to Nogo-A positive cells in spinal cord tissues of patients with ALS (panel b). Human ex vivo cervical spinal cord tissues were stained for ERVK CTXLP (red), TCF4 (green), Nogo-A (grey) and nuclei (blue) in NN controls (n = 5) and patients with ALS (n = 5). Image merging for CTXLP and TCF4 indicate that oligodendrocyte precursors express CTXLP in ALS. White stars indicate areas that are magnified to depict overlapping protein expression in $CTXLP^+$ rings.

Supplementary information

SUPPLEMENTARY METHODS

Bioinformatics

Open reading frames (ORFs) in the ERVK genome were predicted on both the sense and antisense strands using CLCbio software. Any amino acid-encoding codon was accepted as an ORF start, although each ended with a stop codon. All ORFs identified were searched using NCBI Conserved domains (https://www.ncbi.nlm.nih.gov/Structure/cdd/cdd.shtml) and PFAM (https://pfam.xfam.org/). ERVK Env-encoding RNA was inserted into RNAfold software to predict RNA secondary structure (http://rna.tbi.univie.ac.at/cgi-bin/RNAfold.cgi). The likelihood that these ERVK RNA secondary structures represent an internal ribosome entry site (IRES) was determined using IRES prediction software called IRESite (http://iresite.org/IRESite_web.php) and reported as a similarity with known cellular and viral IRES 2D structures. The ERVK conotoxin-like protein (CTXLP) domain was compared to inhibitor cystine knot (ICK) proteins and other cysteine-rich proteins (**Table S1**) using Geneious Prime software[8]. Tertiary structure prediction of the ERVK113 CTXLP domain was performed using Knotter 1D3D software. The structure alignment is based on ERVK CTXLP and nuclear polyhedrosis virus (NPV) CTXLP sequence alignment and was prepared using UCSF Chimera software [3]. Prediction of DNA binding was performed using DNABind software (http://dnabind.szialab.org/). Prediction parameters were as follows: false-positive rate of 6%, expected sensitivity of 58.7%, expected Matthews correlation coefficient of 0.55, the score threshold is set to 1.577 (threshold probability of 0.8288). The identification of CTXLP variants in humans was based on genome build GRCh38. Translated open reading frames longer than 59 bp identified in human ERVK loci annotated by RepeatMasker were searched for PF08087 by HMMER. The matching sequences were clustered using cd-hit.

Supplementary information

RNA seq analysis

To evaluate the significance of CTXLP in disease, we evaluated the expression of CTXLP encoding ERVK loci in publicly available RNA-Seq datasets in the Sequence Read Archive (SRA) (**Figure S6**). These loci were identified by searching the SRA by disease affiliation and then evaluating each potential study based on sample size, tissue, and sequencing quality. Preference was given to studies with large sample sizes, autologous controls, ex vivo disease-relevant tissue, and high sequencing quality. Paired-end reads were preferred to single-end. We focused on studies with fewer measures selecting for particular RNA subpopulations, which could have depleted ERVK RNA from the input. Using the Compute Canada cluster Orcinus, FASTQ files for each run from each study were downloaded from the SRA using fastq-dump, trimming positions for each study were chosen based on examining the output of FastQC, and reads were then mapped to an unmasked copy of hg38 obtained from UCSC using bowtie2. Expression of ERVK loci was measured using samtools view to count reads aligning to each locus, with expression data interpreted using EdgeR. Principle component analysis (PCA) was used to map the clustering of transcript expression in different clinical groups.

Orthologues and paralogues analysis

The most recent genomic assembly for each primate species was searched for CTXLP in the same manner as the human genome. panTro5 and gorGor5 were retrieved from UCSC, and Caty_1.0 was retrieved from NCBI. Chimpanzee ERVK insertions were identified using UCSC table panTro5.nestedRepeats, but no such table exists for the Gorilla or Sooty Mangabey. Gorilla and Mangabey ERVK were identified directly from RepeatMasker output. To reduce the number of small ERV fragments to be BLASTed and to increase the accuracy of orthology predictions by including flanking genomic regions, the loci annotated in RepeatMasker were extended by 1000 bp to either side and then any less than 10 bp apart were merged. MUSCLE alignments of tBLASTx results from loci in

humans, chimpanzee, gorilla, and mangabey where an orthologue in at least one species encodes a Toxin_18+ ORF are reported. Orthology was determined by pairwise best BLAST matches of whole retroelement entries and their flanking 1,000 bp, which mostly correspond to entire ERVs, but which sometimes were fragments. Three and four-way orthology was determined from pairwise orthology. A Heatmap was generated by superheat from frames 0 (CTXLP) and 1 (Envelope) of the human and gorilla orthologs ORFs, which were both positive for Toxin_18 (CTXLP).

Ethics statement

All research involving human autopsy tissue was approved by the University of Winnipeg Human Research Ethics Board under protocol HE-#791 (protocol approval 24 January 2012). Anonymized autopsy ALS ($n = 19$) and neuro-normal control ($n = 18$) tissue specimens were obtained from the NIH NeuroBioBank (USA, https://neurobiobank.nih.gov/) and VA Brain bank (USA, https://www.research.va.gov/programs/specimen_biobanking.cfm).

Diagnosis and demographics of patient samples

Pathologic examination was used to confirm the clinical diagnosis of ALS. The post-mortem interval of all patients was <24 h. **Table S3** indicates the individual patient diagnosis, location of brain tissue sampling, age, gender, and post-mortem interval (PMI in hours) of the samples used in this study. The brain regions analyzed were the motor cortex (Brodmann areas, BA4 & BA6) and cervical and lumbar spinal cord (CC, LC).

Immunohistochemistry of autopsy tissue

To determine the extent of ERVK CTXLP and cellular target expression patterns in the CNS of ALS patients, immunohistochemistry was performed to detect the levels and localization of these target proteins in autopsy human cortical brain tissue, as previously

described [9]. Primary antibodies used are described in **Table S4.** Tissues were also counterstained with DAPI. Free-floating tissues were mounted onto slides and stained in a 0.1% solution of Sudan Black B. Slides were rinsed, and coverslips mounted using ProLong Gold anti-fade reagent (Molecular Probes). All samples were batch stained with case-control matched tissues. Immunostained tissues were imaged with Olympus FV1200 laser scanning confocal microscope fitted with the Olympus Fluoview version 4.0B software suite. This software was used to outline cellular as well as nuclear boundaries of ERVK$^+$ neurons in neuro-normal and ALS specimens. Quantification of micrographs was done using ImageJ to perform the intensity, puncta, and colocalization measurements. GraphPad Prism was used to perform statistical analysis of micrograph data between neuro-normal and ALS patient groups.

Solochrome cyanine staining

Free-floating cervical and lumbar spinal cord sections (60 μm) in ethylene glycol-sucrose solution from human neuro-normal (NN) and amyotrophic lateral sclerosis (ALS) patient tissues (n = 3 for each group and cord region) were used for solochrome cyanine staining. Sections were transferred to well plates and rinsed three times for five minutes with Tris-buffered saline (TBS) and 0.05% Triton X100. After washing, sections were carefully mounted onto Superfrost+ slides and dried before staining. Histochemical staining was performed for solochrome cyanine and counterstaining with eosin for visualization of myelin, as previously described[10], using erichrome cyanine R (J.T. Baker #L128-05) and 10% Iron Alum (ferric ammonium sulfate, Fisher #I75-500) solutions for staining and Eosin Y (Fisher #E511-25) solution for counterstaining. Sections were mounted with Permount, and slides were coverslipped and dried before imaging. Low magnification scanning of slides was performed to obtain whole spinal cord section images of ALS and NN tissue for comparison of myelin staining.

Supplementary information

Cell culture and treatments

The SVGA cell line is derived from immortalized human fetal astrocytes [11] and was maintained in Dulbecco's modified Eagle's medium (DMEM) supplemented with 10% Fetal Bovine Serum (HyClone). ReNcell CX cells (Millipore #SCC007) are immortalized human neural progenitor cells (hNPCs) [12], and were maintained in a proprietary ReNcell neural stem cell medium (Millipore) supplemented with 20 ng/mL human epidermal growth factor (EGF; PeproTech #AF10015) and 20 ng/mL human basic fibroblast growth factor (bFGF; PeproTech #AF10018B). T47D cells were maintained as previously described[13]. All cell lines were maintained in a 37 °C incubator containing 5% CO_2. SVGA cells were seeded into six-well plates and onto glass coverslips at a density of 300,000 cells/mL and 30,000 cells/mll, respectively, for 24 h. To differentiate HNPCs into neurons, ReNcells CX were seeded in laminin (20 μg/mL; Millipore #CC095) coated six-well plates at a density of 50,000 cells/mL for 24 h. Adhered cells were rinsed with 1X PBS and allowed to differentiate in the presence of ReNcell medium lacking growth factors for 10 days.

Immunoprecipitation and cell treatments

Immunoprecipitation (IP) beads (BioRad Surebeads, USA) were prepared by transferring 100 μL of beads into a 1.5 mL Eppendorf tube and washing three times with 1 mL 0.1% PBS-Tween. Next, 10 μL of protein-specific antibody in PBS is added to the tube and incubated for 10 min at room temperature while rotating. The mixture was then centrifuged briefly, and beads are washed three times with 1 mL 0.1% PBS-Tween. Next, 200 μL 0.4% paraformaldehyde was added to beads, and the mixture was incubated for 10 min at room temperature while rotating. Centrifugation and wash steps were repeated. Beads were then incubated for 1 h at room temperature, rotating, with 500 ug of whole-cell extracts in PBS. Centrifugation and wash steps were repeated, and beads were transferred to a new tube before the last wash was removed. Beads were incubated

a final time with 20 μL 0.1M glycine, pH 2.85, for 5 min at room temperature. The supernatant was then transferred into a new 1.5 mL Eppendorf tube containing 2 μL 0.5 M NaOH.

Cells were dosed by volume of IP product (5 μL standard dose), as there was no reliable way to measure the concentration of the protein in the IP product, as protein concentration was vanishingly below the sensitivity of our in-house BCA assay (20 μg/mL).

Immunocytochemistry

For the cleaved caspase-3 assays, SVGA astrocytes were cultured in complete Dulbecco's modified eagle's medium in 12 well plates until 80% cell confluency was reached. SteriFlip Vacuum-driven System Filtration system (50 mL, 0.22 μm pore size, SE1M179M6) was used to prepare sterile cell treatments. Prior to treatment, culture media was removed, and cells were washed twice with phosphate-buffered saline before adding 500 μL of treatment or imaging media per well. This resulted in wells treated with purified protein containing 5 μL of immunoprecipitation (IP) product with or without the addition of 10 mM of $CaCl_2$. Treated cells were then incubated at 37 °C for 24 h before imaging. Next, 20 min prior to imaging, two drops of each NucBlue Live Cell Stain ReadyProbes reagent (Molecular Probes R37605) and CellEvent Caspase-3/7 Green ReadyProbes reagent (Molecular Probes R37111) were added to each well and returned to this incubator until imaging cells. Cells were imaged using an EVOS microscope in the transmission, blue and green fluorescent channels. SVGA cells were treated with 0.1 ng/ml human TNFα or human LIGHT (PeproTech). Untreated cells were used as negative control. Twenty-four hours post-treatment, cells were fixed with methanol for 40 seconds and rinsed with 1X PBS. Cells were permeabilized with 250 μL of PBS-T (PBS with 0.25% TritonX-100) and blocked with 250 μL of 3% BSA in TBS-T (TBS with 0.25% TritonX-100)

for 30 min. Immunocytochemistry was performed using primary antibodies for 3 h and secondary antibodies for 2 h. Nuclei were counterstained with DAPI. Coverslips were mounted onto slides using ProLong Gold anti-fade reagent (Molecular Probes) and dried overnight. Controls were prepared by immunostaining without the primary antibodies. Confocal microscopy was performed using an Olympus FV1200 laser scanning confocal microscope. Olympus Fluoview software was used to outline cellular and nuclear boundaries, with quantification of total intensity (integration) in each compartment reported. GraphPad Prism was used to perform statistical analysis of micrograph data between treatment groups using paired t-tests.

Transient transfections

Empty pcDNA3.1, custom pcDNA3.1-CTXLP, and custom pcDNA3.1-SU vectors were synthesized by GenScript, USA (to be reposited on Addgene). SVGA cells (or 293T cells) were transfected with 2 μg of these plasmids individually using 6 μL of Lipofectamine LTX Reagent, as per manufacturer's instructions (Invitrogen #15338-100). Cells were transfected in serum-free culture media for 4 h, followed by addition of complete media. Cells were harvested 24 h post-transfection. Un-transfected cells and those transfected with the empty vector were used as the negative controls.

Western Blotting

Harvested cells were lysed on ice with 50 μL of in-house lysis buffer (0.05M Tris (pH 7.4), 0.15M NaCl, 0.002M EDTA, 10% glycerol and 1% NP-40 in ultra-pure water) to extract proteins. Pellets remaining from lysis buffer extraction were lysed with 50 μL in-house RIPA buffer (1X TBS, 1% SDS, 1% NP40, and 0.5% DOC in ultra-pure water). Autopsied tissues were lysed on ice with in-house RIPA lysis buffer (1X TBS, 1% SDS, 1% NP40, and 0.5% DOC in ultra-pure water) and homogenized (3 min at a frequency of 30 1/sec) with ceramic beads to extract proteins. All lysis buffer was supplemented with 1× HALT

protease and phosphatase inhibitor cocktail (Thermo Scientific #78442). BCA assay (Thermo Scientific #PI23227) was used to determine the protein content of each sample as per the manufacturer's instructions. Cell lysates were prepared for SDS-PAGE and heated at 95 °C for 10 min. Proteins (15 μg per lane) were separated by SDS-PAGE using a 10% polyacrylamide gel and transferred onto a nitrocellulose membrane. A prerun cancer cell line blot was obtained from GBiosciences (TB-55). Membranes were blocked in 5% skim milk solution for 30 min and probed with the desired primary antibody overnight at 4 °C, followed by incubation at room temperature for 2 h. Primary antibodies used are listed in **Table S4**. The membrane was then probed with horseradish peroxidase (HRP)-conjugated antibody (Bio-Rad, #170-6516 and #170-6515) or fluorescent-conjugated secondary antibody for 2 h at room temperature. To image HRP-conjugated antibodies, the nitrocellulose membrane was developed with 2 ml of Luminata Crescendo Western HRP substrate (Millipore #WBLUR0500) and imaged using Protein Simple FluorChem M chemiluminescent imager. To image fluorescent-conjugated antibodies, low fluorescence polyvinylidene fluoride membrane (Biorad, USA #1620174) was directly imaged using Protein Simple FluorChem M imager. Image Lab software (BioRad, USA) was used to determine the molecular weight and relative density (normalized to β-actin) of each band. GraphPad Prism was used to compare the patient groups through the Mann–Whitney t-test. Correlation analysis was performed using the Pearson rank correlation coefficient.

Quantitative PCR

QPCR was performed on cDNA generated from transfected 293T cells using SYBR Green detection to amplify inflammatory genes, as previously described [14]. Primers were NF-κB p65 F: 5′-TCAATGGCTACACAGGACCA-3′ and R: 5′-CACTGTCACCTGGAAGCAGA-3′, IRF7 F: 5′-TACCATCTACCTGGGCTTCG-3′ and R: 5′-AGGGTTCCAGCTTCACCA-3′, CXCL10 F: 5′-TTCCTGCAAGCCAATTTTGTC-3′ and

R: 5′-TCTTCTCACCCTTCTTTTTCATTGT-3′, Viperin F: 5′-CACAAAGAAGTGTCCTGCTTGGT-3′ and R: 5′-AAGCGCATATATTTCATCCAGAATAAG -3′.

Chromatin immunoprecipitation (ChIP)

SVGAs were seeded in 10 cm dishes at an approximate density of 3 × 10^6 cells/dish for 24 h at 37 °C and 5% CO_2. Laminin-coated dishes were used to seed ReNcell CX cells at a density of 3 × 10^5 cells/dish for 24 h at 37 °C and 5% CO_2. The culture media on adhered ReNcell CX cells was then replaced with that lacking EGF and bFGF growth factors, and cells were allowed to differentiate into neurons for 10 days. SVGAs and neurons were treated with 10 ng/ml human TNFα (PeproTech) or human LIGHT (PeproTech) for 8 h, fixed with 4% paraformaldehyde, and harvested. Untreated cells were used as the negative control. Chromatin Immunoprecipitation (ChIP) was performed using the Pierce Magnetic ChIP kit (Thermo Scientific #26157) as per manufacturer's instructions. CTXLP bound DNA segments were isolated using 5 μg of rabbit anti-CTXLP (custom) antibody. Immunoprecipitation with IgG antibody was used as negative control. QPCR was performed on the immunoprecipitated DNA using SYBR Green detection to amplify the ISREs in the ERVK 5′ LTR. Primers for the first ISRE (nt. 380–392) were F: 5′-TCACCACTCCCTAATCTCAAGT-3′ and R: 5′-TCAGCACAGACCCTTTACGG-3′ and for second ISRE (nt. 563–575) were F: 5′- CTGAGATAGGAGAAAAACCGCCT-3′ and R: 5′-GGAGAGGGTCAGCAGACAAA-3′, as previously described[14]. Data were analyzed using the ΔΔCt method and normalized relative to the input and IgG controls for each condition. All data were graphed as mean ± standard error of measurement. Statistical analyses were performed in GraphPad PRISM using Two-Way ANOVA and Tukey's multiple comparisons test.

Supplementary information

LIST OF ABBREVIATIONS

**ALS**	Amyotrophic lateral sclerosis
**BA 4 & 6**	Brodmann area 4 & 6 (motor cortex)
**BBB**	Blood-brain barrier
**BCA**	Bicinchoninic acid assay
**BMAA**	beta-N-methylamino-L-alanine
**CACNA1B**	Cav2.2
**CC**	Cervical spinal cord
**ChIP**	Chromatin Immunoprecipitation
**CNS**	Central nervous system
**CTXLP**	Conotoxin-like protein
**CX3CL1**	Chemokine (C-X3-C motif) ligand 1
**DAPI**	4′,6-diamidino-2-phenylindole
**DNA**	Deoxyribonucleic acid
**ERVH/K/W**	Endogenous retrovirus-H/-K/-W
**FTD**	Frontotemporal dementia
**HIV**	Human Immunodeficiency Virus
**HIV Tat**	Human Immunodeficiency Virus transactivator of transcription protein
**hNPC**	Human neural progenitor cells
**HRP**	Horseradish peroxidase
**HTLV**	Human T-Lymphotropic Virus
**ICK**	Inhibitor cysteine knot
**IP**	Immunoprecipitation/Immunoprecipitated
**IRES**	Internal ribosomal entry site
**ISRE**	Interferon-stimulated response element
**LC**	Lumbar spinal cord
**LIGHT**	Homologous to lymphotoxin, exhibits inducible expression, and competes with HSV glycoprotein D for herpes virus entry mediator, a receptor expressed by T lymphocytes
**LTR**	Long terminal repeat
**MAG**	Myelin-associated glycoprotein
**MAP2**	Microtubule-associated protein 2
**MBP**	Myelin basic protein
**MLKL**	Mixed lineage kinase domain-like
**MMTV**	Mouse mammary tumor virus
**MOG**	Myelin oligodendrocyte glycoprotein
**mRNA**	Messenger RNA

Supplementary information

**MS**	Multiple Sclerosis
**NF-κB**	Nuclear factor kappa B
**NLS**	Nuclear localization sequence
**NN**	Neuro-normal
**Nogo-A**	Neurite outgrowth inhibitor
**NPV**	Nuclear polyhedrosis virus
**OL**	Oligodendrocyte
**Olig1**	Oligodendrocyte transcription factor 1
**Olig2**	Oligodendrocyte transcription factor 2
**OPC**	Oligodendrocyte precursor cell
**ORF**	Open reading frame
**PBS**	Phosphate buffered saline
**PCA**	Primary component analysis
**PFAM**	Protein family
**PLP**	Myelin proteolipid protein
**PMI**	Post-mortem interval
**PRF**	Programmed ribosomal frameshifting
**RIP1**	Receptor-interacting protein kinase 1
**RIP3**	Receptor-interacting protein kinase 3
**RNA**	Ribonucleic acid
**RPMI**	Roswell Park Memorial Institute
**RT**	Reverse transcriptase
**SC**	Solochrome cyanine
**SRA**	Sequence read archive
**SU**	Surface unit
**TCF4**	Transcription factor 4
**TM**	Transmembrane
**TNFα**	Tumor necrosis factor alpha
**tRNA**	Transfer RNA
**VGCC**	Voltage-gated Calcium channels
**WCE**	Whole cell extract

SUPPLEMENTARY REFERENCES

1. Dinkel, H.; Van Roey, K.; Michael, S.; Kumar, M.; Uyar, B.; Altenberg, B.; Milchevskaya, V.; Schneider, M.; Kuhn, H.; Behrendt, A.; et al. ELM 2016—Data update and new functionality of the eukaryotic linear motif resource. *Nucleic Acids Res.* **2016**, *44*, D294–300, doi:10.1093/nar/gkv1291.
2. Gracy, J.; Le-Nguyen, D.; Gelly, J.C.; Kaas, Q.; Heitz, A.; Chiche, L. KNOTTIN: The knottin or inhibitor cystine knot scaffold in 2007. *Nucleic Acids Res.* **2008**, *36*, D314–319, doi:10.1093/nar/gkm939.
3. Pettersen, E.F.; Goddard, T.D.; Huang, C.C.; Couch, G.S.; Greenblatt, D.M.; Meng, E.C.; Ferrin, T.E. UCSF Chimera—A visualization system for exploratory research and analysis. *J. Comput. Chem.* **2004**, *25*, 1605–1612, doi:10.1002/jcc.20084.
4. Subramanian, R.P.; Wildschutte, J.H.; Russo, C.; Coffin, J.M. Identification, characterization, and comparative genomic distribution of the HERV-K (HML-2) group of human endogenous retroviruses. *Retrovirology* **2011**, *8*, 90, doi:10.1186/1742-4690-8-90.
5. Li, W.; Lee, M.H.; Henderson, L.; Tyagi, R.; Bachani, M.; Steiner, J.; Campanac, E.; Hoffman, D.A.; von Geldern, G.; Johnson, K.; et al. Human endogenous retrovirus-K contributes to motor neuron disease. *Sci. Transl. Med.* **2015**, *7*, 307ra153, doi:10.1126/scitranslmed.aac8201.
6. Gao, B.; Peng, C.; Yang, J.; Yi, Y.; Zhang, J.; Shi, Q. Cone Snails: A Big Store of Conotoxins for Novel Drug Discovery. *Toxins* **2017**, *9*, doi:10.3390/toxins9120397.
7. Brohawn, D.G.; O'Brien, L.C.; Bennett, J.P., Jr. RNAseq Analyses Identify Tumor Necrosis Factor-Mediated Inflammation as a Major Abnormality in ALS Spinal Cord. *PLoS ONE* **2016**, *11*, e0160520, doi:10.1371/journal.pone.0160520.
8. Kearse, M.; Moir, R.; Wilson, A.; Stones-Havas, S.; Cheung, M.; Sturrock, S.; Buxton, S.; Cooper, A.; Markowitz, S.; Duran, C.; et al. Geneious Basic: an integrated and extendable desktop software platform for the organization and analysis of sequence data. *Bioinformatics* **2012**, *28*, 1647–1649, doi:10.1093/bioinformatics/bts199.
9. Douville, R.; Liu, J.; Rothstein, J.; Nath, A. Identification of active loci of a human endogenous retrovirus in neurons of patients with amyotrophic lateral sclerosis. *Ann. Neurol.* **2011**, *69*, 141–151, doi:10.1002/ana.22149.
10. Di Curzio, D.L.; Buist, R.J.; Del Bigio, M.R. Reduced subventricular zone proliferation and white matter damage in juvenile ferrets with kaolin-induced hydrocephalus. *Exp. Neurol.* **2013**, *248*, 112–128, doi:10.1016/j.expneurol.2013.06.004.
11. Major, E.O.; Miller, A.E.; Mourrain, P.; Traub, R.G.; de Widt, E.; Sever, J. Establishment of a line of human fetal glial cells that supports JC virus multiplication. *Proc. Natl. Acad. Sci. USA* **1985**, *82*, 1257–1261.
12. Donato, R.; Miljan, E.A.; Hines, S.J.; Aouabdi, S.; Pollock, K.; Patel, S.; Edwards, F.A.; Sinden, J.D. Differential development of neuronal physiological responsiveness in two human neural stem cell lines. *BMC Neurosci.* **2007**, *8*, 36, doi:10.1186/1471-2202-8-36.
13. Golan, M.; Hizi, A.; Resau, J.H.; Yaal-Hahoshen, N.; Reichman, H.; Keydar, I.; Tsarfaty, I. Human endogenous retrovirus (HERV-K) reverse transcriptase as a breast cancer prognostic marker. *Neoplasia* **2008**, *10*, 521–533.

14. Manghera, M.; Ferguson-Parry, J.; Lin, R.; Douville, R.N. NF-kappaB and IRF1 Induce Endogenous Retrovirus K Expression via Interferon-Stimulated Response Elements in Its 5' Long Terminal Repeat. *J. Virol.* **2016**, *90*, 9338–9349, doi:10.1128/JVI.01503-16.
